# Anti-PD-L1 antibody ASC22 in combination with a histone deacetylase inhibitor chidamide as a “shock and kill” strategy for ART-free virological control: a phase II single-arm study

**DOI:** 10.1038/s41392-024-01943-9

**Published:** 2024-09-09

**Authors:** Luling Wu, Zhihang Zheng, Jingna Xun, Li Liu, Jiangrong Wang, Xinyu Zhang, Yueming Shao, Yinzhong Shen, Renfang Zhang, Min Zhang, Meiyan Sun, Tangkai Qi, Zhenyan Wang, Shuibao Xu, Wei Song, Yang Tang, Bihe Zhao, Zichen Song, Jean-Pierre Routy, Hongzhou Lu, Jun Chen

**Affiliations:** 1 Department of Infectious Diseases and Immunology, Shanghai Public Health Clinical Center, Fudan University, Shanghai, China; 2 Institute of Antibiotics, Huashan Hospital, Fudan University, Shanghai, China; 3 Shanghai Institute of Infectious Disease and Biosecurity, Fudan University, Shanghai, China; 4 State Key Laboratory of Genetic Engineering and Engineering Research Center of Gene Technology, Ministry of Education, Institute of Genetics, School of Life Sciences, Fudan University, Shanghai, China; 5 Department of Clinical Laboratory, Shanghai Public Health Clinical Center, Fudan University, Shanghai, China; 6 Infectious Disease and Immunity in Global Health Program, Research Institute of McGill University Health Centre, Montreal, QC Canada; 7 Department of Infectious Diseases and Nursing Research Institution, National Clinical Research Center for Infectious Diseases, The Third People’s Hospital of Shenzhen, Guangdong, China

**Keywords:** Clinical trials, Infectious diseases

## Abstract

The combination of ASC22, an anti-PD-L1 antibody potentially enhancing HIV-specific immunity and chidamide, a HIV latency reversal agent, may serve as a strategy for antiretroviral therapy-free virological control for HIV. People living with HIV, having achieved virological suppression, were enrolled to receive ASC22 and chidamide treatment in addition to their antiretroviral therapy. Participants were monitored over 24 weeks to measure changes in viral dynamics and the function of HIV-specific CD8^+^ T cells (NCT05129189). 15 participants completed the study. At week 8, CA HIV RNA levels showed a significant increase from baseline, and the values returned to baseline after discontinuing ASC22 and chidamide. The total HIV DNA was only transiently increased at week 4 (*P* = 0.014). In contrast, integrated HIV DNA did not significantly differ from baseline. Increases in the proportions of effector memory CD4^+^ and CD8^+^ T cells (T_EM_) were observed from baseline to week 24 (*P* = 0.034 and *P* = 0.002, respectively). The combination treatment did not succeed in enhancing the function of HIV Gag/Pol- specific CD8^+^ T cells. Nevertheless, at week 8, a negative correlation was identified between the proportions of HIV Gag-specific T_EM_ cells and alterations in integrated DNA in the T cell function improved group (*P* = 0.042 and *P* = 0.034, respectively). Nine adverse events were solicited, all of which were graded 1 and resolved spontaneously. The combined treatment of ASC22 and chidamide was demonstrated to be well-tolerated and effective in activating latent HIV reservoirs. Further investigations are warranted in the context of analytic treatment interruption.

## Introduction

Combination antiretroviral therapy (ART) significantly improves the prognosis for people living with HIV (PLWH) by effectively reducing the plasma human immunodeficiency virus (HIV) viral load to undetectable levels.^1,2^ Despite of effective ART, HIV may persist in both latent and transcriptionally active states, within quiescent or proliferating cells, across various T cell sub-populations encompassing defective and intact viruses.^3^ These T cells constitute a potential HIV latent reservoir that has evaded immune detection and remained unresponsive to ART.^4^ HIV reactivation from latency after ART treatment interruption occurs, on average, every 5–8 days, which has implications for HIV remission. Therefore, innovative therapeutic approaches targeting persistent HIV reservoirs are necessary to achieve an ART-free virological control.^5,6^ To date, latency-reversing agents evaluated in HIV-infected individuals receiving ART, such as histone deacetylase (HDAC) inhibitors and toll-like receptor agonists alone, have failed to consistently demonstrate latency reversal.^7^ No intervention other than allogeneic stem cell transplantation has shown sustained reduction or elimination of cells containing replication-competent viruses.^8^ The most in-depth investigated HIV eradication is the “shock and kill” strategy, which seeks to activate the latent reservoir with latency reversing agents (LRAs) and promote elimination through viral cytopathic effects or immune-mediated clearance.^9^

PD-1, a transmembrane immune receptor belonging to the CD28 family, has been identified as a crucial negative regulator of T cell function.^10^ It interacts with its ligand, programmed death ligand 1 (PD-L1), which is widely expressed on T cells, B cells, dendritic cells, and macrophages.^11^ Envafolimab (ASC22), a humanized anti-PD-L1 antibody derived from camel single domains and used with a human immunoglobulin Fc fragment to extend its half-life, is approved for treating advanced solid tumors.^12–14^Recently, anti-programmed cell death protein 1/programmed death ligand 1 (PD-1/PD-L1) checkpoint inhibitors have drawn considerable attention for their potential for cancer and persistent viral infections therapeutic applications.^10,11,15^ Numerous experiments both in vivo and in vitro have shown that elevated levels of antigenemia following HIV infection can lead to impaired T cell function, mediated by PD-1 and its ligands via immunomodulatory signaling pathways, as evidenced by studies in HIV-infected humanized mice.^16–18^ T cell exhaustion features multiple deficiencies in effector functions, such as impaired proliferation, cytotoxicity, and cytokines production. These deficiencies contribute to weakening the immune system’s capacity to control HIV viral infections. Recent researches indicate that inhibiting the interaction between PD-1 and its ligand, PD-L1 can partially restore T cell exhaustion.^19,20^ Furthermore, there is evidence suggesting that using anti-PD-1/PD-L1 antibody compounds may effectively activate HIV viral expression in latently infected CD4^+^ T cells.^21^ Immune checkpoint inhibitors (ICIs) have the capability to enhance the clearance of cells harboring latent infections, offering a promising approach towards achieving ART-free virological control for HIV infection.^5,22,23^ Nonetheless, the lack of PLWH in a substantial number of clinical trials has restricted our understanding of the safety and durability of HIV-targeted effects associated with immune checkpoint inhibitor therapies in this population. A few reports of ART-treated PLWH with tumors receiving anti-PD-1 therapy have documented transient increases in cell-associated (CA) HIV RNA or plasma HIV RNA, suggesting that immune checkpoint inhibition may impact the reservoir of latent HIV.^24,25^ Additionally, small-scale studies on HIV-associated tumors have demonstrated favorable safety profiles for combined treatment with ICIs.^7,26^ However, there is still lacking of clinical trials assessing the efficacy of anti-PD-1/PD-L1 inhibitors for achieving ART-free virological control.

Various latency-reversing agents (LRAs) have been developed to target different HIV transcriptional regulatory pathways. These mainly include PKC agonists, atypical NF-κB agonists, and HDAC inhibitors (HDACis).^27^ The regulation of HDAC activity on the HIV long terminal repeat (LTR) promoter has been extensively documented as critical for enhancing HIV expression.^28,29^ Chidamide, a HDAC inhibitor in the benzamide category, is specifically designed for oral administration to inhibit HDAC1, 2, 3, and 10 selectively and was approved by the China Food and Drug Administration (CFDA) in December 2014 for treating relapsed or refractory PTCL. Chidamide has also proven effective in enhancing the cytotoxic response of immune cells.^30,31^ Recent studies have revealed a property of chidamide as a HIV latency reversing agent, capable of reactivating latent HIV in primary CD4^+^ T cells.^28,32^ In vivo researches have shown the safe and effective disruption of HIV-1 latency by chidamide, indicating its potential as a promising candidate for clinical investigation in activating the HIV viral reservoir.^33^ Several studies indicate that HDAC inhibitors can rapidly reduce PD-L1 expression at the surface of cells across various cancers, enhancing the anti-tumor efficacy of ICIs and facilitating activated T cell infiltration into tumor microenvironments.^34,35^ Therefore, ASC22 may synergize with chidamide in activating the HIV reservoir.

Collectively, we hypothesize that treatment with the combination of ASC22 and chidamide may potentially enhance the removal of infected cells and bolster the immunity of HIV specific CD8^+^ T cell, resulting in ART-free viral control. During this phase II clinical trial, our objective was to assess the effectiveness and safety of using the combination of ASC22 and chidamide as a “shock and kill” strategy for ART-free virological control in PLWH.

## Results

### Baseline characteristics of the participants

**Fig. 1:**
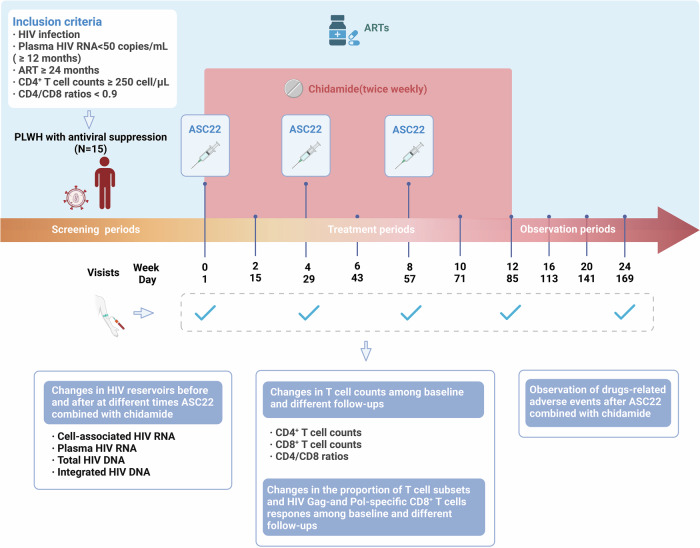
Flowchart of specimens collection and research protocol in relation to ASC22 combined with chidamide administration. The duration of ART treatment is indicated by blue shading, while the duration of chidamide treatment is indicated by red shading. Figure 1 was created with biorender.com. ART antiviral therapy

From July 1, 2022 to March 6, 2023, 18 participants were screened, and 3 individuals were considered ineligible for various reasons (Supplementary Fig. 1). These included having a plasma HIV viral load exceeding 50 copies/mL, experiencing renal insufficiency, or refusing to adhere to the prescribed medication. All 15 enrolled participants completed the clinical trial (Fig. 1 and Table 1). The study comprised solely male participants of Asian ethnicity. Their median age was 36 years, with a median duration of ART was 61.27 months (Interquartile range (IQR), 25–174.17 months). At baseline, the median CD4^+^ T cell counts and the ratios of CD4/CD8 were 433 cells/*μ*L (IQR, 348–730 cells/*µ*l) and 0.6 (IQR, 0.52–0.74), respectively.

**Table 1:** Participant characteristics

No.	Age (years)	Gender	Ethnicity	BMI (kg /m^2^)	Months since HIV diagnosis	Days from HIV diagnosis to ART initiation	Months on ARTs	Current ART regimens	Months with HIV RNA < 50 copies/mL	Baseline CD4 counts (cells/*μ*L)	Baseline CD8 counts (cells/*μ*L)	Baseline CD4/CD8 ratios
01	29	Male	Asian	20.6	52.1	0	52.1	ABC, 3TC, DTG	45.3	304	571	0.5
02	28	Male	Asian	19.2	158.1	2570	72.5	TAF, 3TC, DTG	32.8	539	814	0.7
03	53	Male	Asian	28.1	39	19	38.4	ABC, 3TC, DTG	33.4	370	879	0.4
04	25	Male	Asian	21.9	35.9	251	27.6	TDF, 3TC, EFV	21.8	405	670	0.6
05	30	Male	Asian	22.7	57.1	54	55.3	TDF, 3TC, EFV	49.6	433	578	0.8
06	38	Male	Asian	30.0	111	1291	68	TDF, 3TC, EFV	49.2	633	1355	0.5
08	51	Male	Asian	25.1	103.6	197	97	ABC, 3TC, DTG	49.8	902	1462	0.6
09	34	Male	Asian	28.1	57.6	18	57	TAF, FTC, DTG	50.9	785	998	0.8
10	31	Male	Asian	25.0	62.1	26	61.3	TDF, 3TC, LPV/r	30.5	730	1319	0.6
11	33	Male	Asian	22.5	85	1311	41.3	TDF, 3TC, EFV	35.7	844	989	0.9
12	53	Male	Asian	18.4	144.5	23	143.7	TDF, 3TC, EFV	98.4	284	547	0.5
13	42	Male	Asian	26.9	25	0	25	TDF, 3TC, EFV	24.8	670	991	0.7
14	36	Male	Asian	21.7	75.6	41	74.2	TDF, 3TC, EFV	69	398	712	0.6
15	37	Male	Asian	20.7	198.7	735	174.2	AZT, 3TC, EFV	102.9	349	1074	0.3
16	38	Male	Asian	20.8	87.5	163	82.1	TDF, 3TC, EFV	76.3	327	444	0.7
Median (range)	36 (25–53)			22.5 (20.7–26.9)	75.6 (25–198.7)	54 (0–2570)	61.3 (25–174.1)		49.2 (21.8–102.9)	433 (284–902)	879 (444–1462)	0.6 (0.3–0.9)

Continuous variables are presented as median [interquartile range, (IQR)]

*ABC* Abacavir, *ART* antiviral therapy, *AZT* azidothymidine, *BMI* body mass index, *DTG* Dolutegravir, *TAF* tenofovir alafenamide fumarate, *TDF* tenofovir disoproxil fumarat, *EFV* Efavirenz, *LPV/r* lopinavir/ritonavir, *3TC* lamivudine

### ASC22 and chidamide potentiates HIV latency reversal

**Fig. 2:**
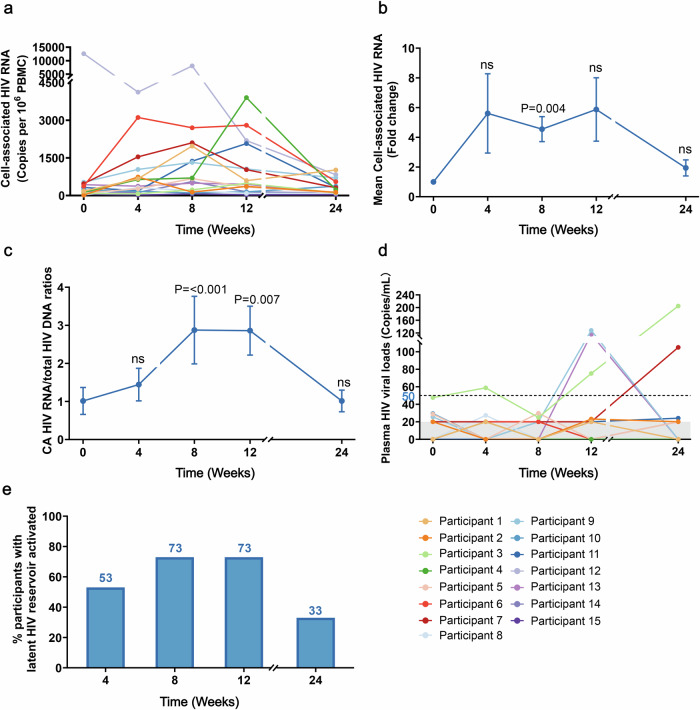
ASC22 combine with chidamide treatment and latency reversal. **a** Changes in cell-associated (CA) HIV RNA levels at weeks 0 (baseline), 4, 8, 12, and 24 for each individual. **b** Mean fold increase in CA HIV RNA levels from baseline. Changes in the ratios of CA HIV RNA to total HIV DNA (**c**) and individual dynamics of plasma HIV viral loads (**d**) at weeks 0 (baseline), 4, 8, 12, and 24. The black dotted line represents the plasma HIV RNA less than 50 copies/ml, and the gray shade represents the plasma HIV RNA less than 20 copies/ml. **e** Proportion of participants with activated latent HIV-1 reservoirs across different treatment cycles and observation periods. Activation of the latent HIV reservoir is indicated by either a greater than twofold increase in CA HIV RNA or plasma HIV viral loads exceeding the threshold of 50 copies/ml. ns means not significant

After administering ASC22 and chidamide, a gradual elevation in the levels of CA RNA: total HIV DNA ratios were observed from baseline to week 4 (Fig. 2a–c, Supplementary Figs. 2a, 3). At week 8, CA HIV RNA levels showed a significant increase from baseline, with an average rise of 4.27-fold (*P* = 0.004). The CA HIV RNA exhibited a gradual downward trend at week 8, returning to baseline levels by week 24. Interestingly, The CA HIV RNA: total DNA ratios were significantly higher at weeks 8 and 12 compared to baseline, showing an increase of 1.87-fold and 2.14-fold, respectively (*P* < 0.001, *P* = 0.007). At week 24, the ratios of CA-RNA to total HIV DNA returned to baseline. No significant difference in plasma HIV viral loads were detected at both weeks 4 and 8 compared to the baseline. However, at week 12, the plasma HIV viral loads increased in three participants. Plasma HIV RNA levels of 105 copies/ml and 205 copies/ml were observed in two participants at week 24, respectively. Only one of these four participants had sustained HIV viral loads elevation at both weeks 12 and 24 (Fig. 2d). The proportion of participants exhibiting HIV reservoir activation increased gradually, observed in 53% (8/15), 73% (11/15), and 73% (11/15) of participants at weeks 4, 8, and 12, respectively (Fig. 2e). By week 24, this had dropped to 33% (5/15).

### Combination of ASC22 and chidamide did not reduce the HIV reservoir size

**Fig. 3:**
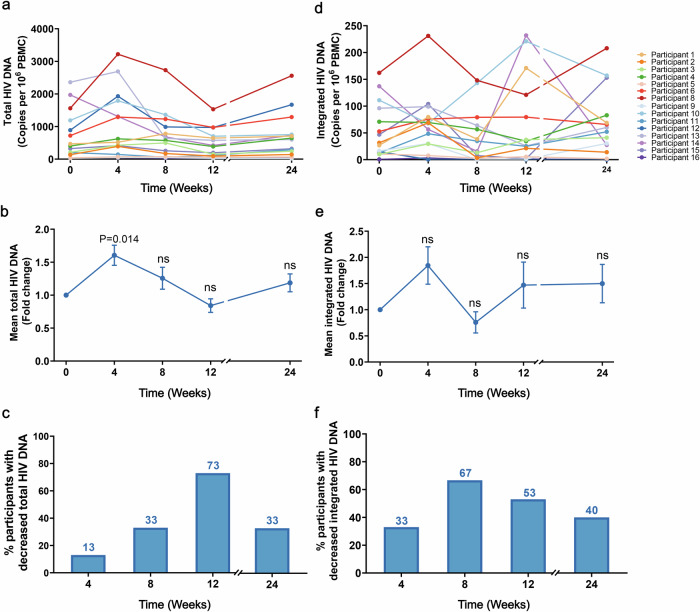
ASC22 combined with chidamide treatment and HIV DNA. **a** Changes in total HIV DNA levels at weeks 0 (baseline), 4, 8, 12, and 24. **b** Mean fold change in total HIV DNA from baseline. **c** The proportion of participants exhibiting a reduction in total HIV DNA levels was measured during both treatment and observation periods. **d** Alterations in integrated HIV DNA at weeks 0 (baseline), 4, 8, 12, and 24 in diverse participants. **e** Mean fold increase in integrated HIV DNA from baseline. **f** The histograms represent the proportion of participants with decreasing integrated HIV DNA levels assessed across various treatment and observation periods. ns means not significant

In this cohort, we did not observe a significant decrease in total or integrated HIV DNA. Compared with the baseline, the total HIV DNA exhibited an increasing trend at week 4 (*P* = 0.014), but subsequently demonstrated a declining trajectory at week 8, ultimately returning to baseline levels by week 12 and week 24 (Fig. 3a, b, Supplementary Figs. 2b, 3). As the duration of therapy prolonged, the proportion of participants experiencing a decrease in total HIV DNA levels gradually increased. This proportion reached 13% (2/15) at week 4, further climbed to 33% (5/15) at week 8, and peaked at 73%(11/15) by week 12. However, by week 24, the proportion regressed to 33% (5/15) (Fig. 3c). The levels of integrated HIV DNA did not significantly change from baseline, despite the activation of the HIV reservoir (*P* = 0.114) (Fig. 3d, e, Supplementary Figs. 2c,3). However, at week 4, 33% (5/15) of participants exhibited a composite decline in integrated HIV DNA, which increased to 67% (10/15) at week 8. At weeks 12 and 24, 53% (8/15) and 40% (6/15) of participants showed a decrease in integrated HIV DNA (Fig. 3f).

### Combination of ASC22 and chidamide raised the proportion of effector memory CD4^+^ and CD8^+^ T cells (T_EM_)

The CD4^+^ T cells primarily included central memory (T_CM_), T_EM_, and naïve (T_N_) subsets, whereas the CD8^+^ T cells predominantly consisted of T_EM_, terminally differentiated memory (T_EMRA_), and T_N_ (Supplementary Figs. 4, 5a). The percentage of CD4^+^ and CD8^+^ T_EM_ cell subsets tended to increase with prolonged treatment, showing significant elevation compared to their respective baseline levels at week 24 (*P* = 0.034; *P* = 0.002) (Supplementary Fig. 4a). In contrast, CD4^+^ T_N_ displayed an overall declining trend, especially at week 24 when compared with the baseline levels (*P* = 0.04) (Supplementary Fig. 4b). The ratios of CD8^+^ T_N,_ CD4^+^ and CD8^+^ T_CM_ and T_EMRA_ cells subsets did not show significant changes during the treatment and observation periods (Supplementary Fig. 4b, c, d). CD4^+^ and CD8^+^ T cell counts, as well as CD4/CD8 ratios in blood remained stable throughout the entire period (Supplementary Fig. 5b–d).

### Combination of ASC22 and chidamide did not improve HIV Gag- and Pol-specific CD8^+^ T cells responses significantly

**Fig. 4:**
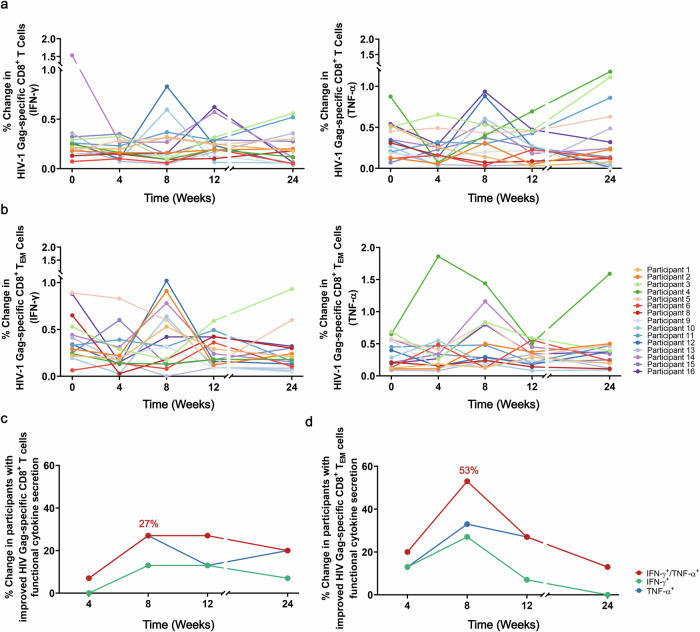
Impact of combination therapy involving ASC22 and chidamide on the responses of HIV Gag-specific CD8^+^ T cells. **a** Individual dynamics of HIV Gag-specific CD8^+^ T cells responses for each participant at weeks 0 (baseline), 4, 8, 12, and 24, indicated for IFN-γ (left) and TNF-α (right). **b** Individual dynamics of HIV Gag-specific CD8^+^ T_EM_ cells responses at weeks 0 (baseline), 4, 8, 12, and 24 for each participant shown for IFN-γ (left) and TNF-α (right). **c**, **d** Changes in the ratios of participants with improvement in HIV Gag-specific CD8^+^ T cells (left) and CD8^+^ T_EM_ cells (right) secreting functional cytokines from baseline to week 24. Improvement in T cell immune function was defined as a more than 2-fold increase in the expression of IFN-γ or TNF-α in HIV Gag- specific CD8^+^ T cells

Throughout both treatment and observation phases, the capacity of HIV Gag-specific CD8^+^ T cells was not enhanced by the combined administration of multiple treatment cycles. However, the functional cytokine secretion by HIV Gag-specific CD8^+^ T cells and CD8^+^ T_EM_ cells gradually increased as the treatment progressed (Fig. 4a, b). At week 8, the ratios of participants exhibiting functional improvement reached their highest level, with rates of 27% (4/15) and 53% (8/15) observed for HIV Gag-specific CD8^+^ T cells and CD8^+^ T_EM_ cells, respectively (Fig. 4c, d). The percentages gradually declined, and at week 24, 20% (3/15) and 13% (2/15) of participants sustained elevated levels of TNF-α secretion by HIV Gag-specific CD8^+^ T cells and CD8^+^ T_EM_ cells, respectively. Out of the participants, only 7% (1/15) demonstrated a sustained elevation above baseline in HIV Gag-specific CD8^+^ T cell functionality. All individuals in the study were observed to have their HIV Gag-specific CD8^+^ T_EM_ cell functionality return to baseline. Similarly, no enhancement in the functionality of HIV Pol-specific CD8^+^ T cells and CD8^+^ T_EM_ cells was observed (Supplementary Fig. 6). Compared to baseline levels, there were no significant alterations observed in PD-1 fluorescence expression on CD4^+^ and CD8^+^ T cells or T_EM_ subset.(Supplementary Fig. 7).

### Subgroup with boosted T cell functions tend to have more pronounced reduction in HIV reservoir size compared to those with unboosted T cell function

**Fig. 5:**
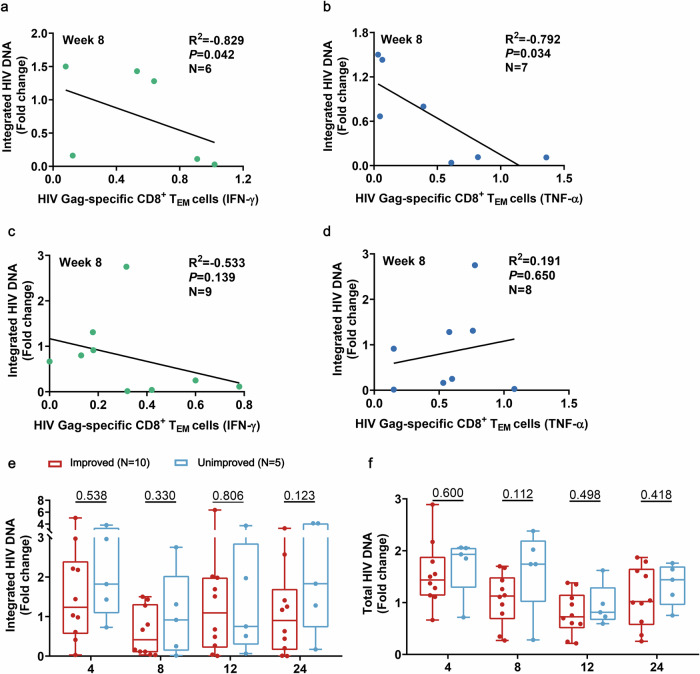
Correlation between fold change of integrated HIV DNA and expression of IFN-γ (left) and TNF-α (right) in the improved (**a**, **b**) and unimproved (**c**, **d**) HIV Gag-specific CD8^+^ T_EM_ cells response group at week 8. Comparison of integrated HIV DNA (**e**) and total HIV DNA (**f**) in T cell function improved and unimproved groups at weeks 4, 8, 12 and 24

At week 8, we observed that the proportion of participants exhibiting T cell functional improvement and a decline in integrated HIV DNA reached a peak. Subsequently, we performed a correlation analysis to investigate further. In the subgroup with T cell function improvement, a negative correlation was detected between the proportions of IFN-γ/TNF-α secreting HIV Gag-specific CD8^+^ T_EM_ cells and alterations in integrated HIV DNA at week 8 (*P* = 0.04, R^2^ = −0.89 and *P* = 0.03, R^2^ = −0.79, respectively) (Fig. 5a, b). No correlation was observed between the expression of IFN-γ/TNF-α in the group with unimproved HIV Gag-specific CD8^+^ T_EM_ cell responses and alterations in integrated HIV DNA (*P* = 0.14, R^2^ = −0.53 and *P* = 0.65, R^2^ = 0.19, respectively) (Fig. 5c, d). Additionally, integrated HIV DNA showed no significant correlation with weak HIV Gag-specific CD8^+^ T cell responses, such as in weeks 4, 12, and 24 (data not shown). At weeks 8 and 24, integrated HIV DNA and total HIV DNA were lower in the T cell function improved group compared to the T cell function unimproved group, although these differences did not observed statistical significance (Fig. 5e, f).

### Combination of ASC22 and chidamide represented safety in HIV virologically suppressed participants

A total of 18 AEs were reported, 9 were classified as solicited systemic AEs, and the remaining 9 were unsolicited (Table 2). Among these, 7 were immune-related adverse events (irAEs), including drug eruption, blood thyroid stimulating hormone decreased, hypertriglyceridemia and liver function test abnormal, which may typically associated with ASC22. Overall, 46.67% (7/15) participants reported at least one AE and 6.67% (1/15) of participants reported more than one solicited AE. All drug-related AEs were grade 1 and resolved spontaneously. Two participants developed blood thyroid stimulating hormone decreased, which recovered without adjusting the dose of ASC22 and chidamide. After receiving the initial dose, one participant experienced drug eruption on the trunk and extremities, which resolved within 24 h. Another individual experienced generalized rash 16 days after dosing, which spontaneously resolved by day 13. Additionally, a different participant developed generalized rash 22 days after receiving the initial dose, which persisted for 5 days and then resolved spontaneously. After the completion of ASC22, one participant developed bilateral lower extremity edema while receiving chidamide alone, which self-recovered without adjusting the dose.

**Table 2:** Adverse events (AEs)

Type of AEs	Grade of adverse events (*N* = 15)					No. of participants
	1	2	3	4	5	
Drug eruption	3 (20)	0 (0)	0 (0)	0 (0)	0 (0)	Participants 2, 12, 15
Gravitational edema	1 (6.67)	0 (0)	0 (0)	0 (0)	0 (0)	Participant 2
Blood thyroid stimulating hormone decreased	2 (13.33)	0 (0)	0 (0)	0 (0)	0 (0)	Participants 1,2
Coronavirus disease 2019 (COVID-19)	4 (26.67)a	0 (0)	0 (0)	0 (0)	0 (0)	Participants 1,2
Nephrolithiasis	1 (6.67)a	0 (0)	0 (0)	0 (0)	0 (0)	Participants 1,4
Weight increased	1 (6.67)a	0 (0)	0 (0)	0 (0)	0 (0)	Participant 2
Hepatic steatosis	1 (6.67)a	0 (0)	0 (0)	0 (0)	0 (0)	Participant 2
Injury	1 (6.67)a	0 (0)	0 (0)	0 (0)	0 (0)	Participant 11
Chest discomfort	1 (6.67)	0 (0)	0 (0)	0 (0)	0 (0)	Participant 9
Blood creatine phosphokinase abnormal	1 (6.67)a	0 (0)	0 (0)	0 (0)	0 (0)	Participant 15
Hypertriglyceridemia	1 (6.67)	0 (0)	0 (0)	0 (0)	0 (0)	Participant 4
Liver function test abnormal	1 (6.67)	0 (0)	0 (0)	0 (0)	0 (0)	Participant 11

1 a represented unsolicited systemic AEs

## Discussion

To our knowledge, the study represents the first prospective cohort investigation involving PLWH who have received a combination of ICI and HDAC inhibitors, with the aim of comprehensively assessing the reversal of HIV latency and immune function in individuals under ART suppression. The simultaneous administration of ASC22 and chidamide over 12 weeks was safe and well-tolerated. Importantly, it can effectively activate latent HIV reservoirs and promote the differentiation of CD4^+^ and CD8^+^ T_N_ cells into functional T_EM_ cells. In the subgroup displaying stronger T cell function, the combination treatment strategy results in a more pronounced reduction in the HIV reservoir. These findings also tend to have a small size of HIV reservoir in populations with boosted T cell function compared with those without T cell functional improvement.

The “shock and kill” strategy, a promising approach for achieving ART-free virological control, harnesses the immune response to target and clear latent HIV infections by selectively inducing the reactivation of latent HIV transcription while avoiding full activation of T cells.^36,37^ During our clinical trial, we noted a substantial rise in CA HIV RNA relative to baseline upon treatment with ASC22 and chidamide. Furthermore, we observed a notable increase in the ratios of CA HIV RNA to total HIV DNA, and some participants displayed an elevation in their peripheral plasma HIV viral loads, indicating a marked activation of the HIV reservoir. Furthermore, levels of the CA HIV RNA returned to the baseline in 67% (10/15) of the participants after discontinuation of ASC22 and chidamide, providing further evidence that the observed changes in HIV transcription were a result of the synergistic effect of the combination therapy strategy. However, due to combined treatment, we cannot distinguish whether the therapeutic effect is solely attributed to this combination or if similar effects would have been observed with either drug alone. Four participants remained that did not respond to during treatment. Since the level of reversal of the latent reservoir is also correlated with its size, in these participants who are difficult to reverse the latent reservoir may have been smaller. At the same time, with long-term ART therapy, the reservoir may become more difficult to reverse, and more research is needed to confirm this. During the treatment periods, we identified a disconnect between CA HIV RNA and plasma HIV viral loads, like in another study.^33^ We speculate that fluctuations in HIV viral loads were not detected due to relatively long monitoring intervals. In our study, during the treatment periods, we identified a disconnect between CA HIV RNA and plasma HIV viral loads, like in another study.^33^ We speculate that fluctuations in HIV viral loads were not detected due to relatively long monitoring intervals. In our study, plasma HIV viral load rebounded in four participants after discontinuation of drug treatment. However, the underlying mechanism behind this phenomenon remains unclear. To explore the potential impact of ASC22 and chidamide on clonal expansion, we endeavored to perform HIV deep sequencing at both baseline and during the rebound of HIV plasma load. Regrettably, we were unable to obtain sufficient HIV sequence information in the plasma samples, likely attributable to the low viral load present in the plasma.

The HIV latent reservoir is commonly believed to evade immune surveillance. Should there be a robust immune response upon HIV reactivation, it could potentially shift the balance in favor of the host in controlling the virus.^38^ However, a sustained decrease in the latent HIV reservoir size was not shown following treatment with ASC22 and chidamide. The failure to adequately eliminate the HIV reservoir may be attributed to immunological recognition impairments in CD8^+^ T cells function.^39,40^ Despite the observed increase in CD4^+^ and CD8^+^ T_EM_ cells, the present study reveals a notable absence of substantial enhancement in the function of HIV-specific CD8^+^ T cells. Firstly, the infection with HIV reduces phosphatase and tensin homolog (PTEN) levels.^41^ Prior oncology investigations revealed that constitutive PD-L1 expression due to PTEN loss might lead to resistance to other cancer immunotherapy approaches by actively suppressing antitumor T cells.^42,43^ Hence, we hypothesized that the lack of PD-L1 response may be due to the decrease in PTEN levels after HIV infection. Secondly, other research has shown the developmental biology of exhausted CD8^+^ T cells (T_ex_) may contribute to the restoration of T cell function.^44^ T cells converted the T_ex_ subset in the third stage of exhaustion, and T cells demonstrated a partial restoration of their functionalities after PD-L1 blockade.^45^ However, once the T cells progress into the terminal exhaustion stage, they exhibit unresponsiveness to PD-L1 blockade.^46^ Thirdly, promoting immune-mediated mechanisms like boosting cytotoxic T cell responses can achieve prolonged viral control, contingent upon continual viral replication and the expression of viral antigens.^47^ Previous studies in non-human primates indicate that achieving viral control may require a phase of rebound viremia after ART interruption (ATI); PD-1 blockade administered post-ATI has been shown to significantly enhance the functionality of CD8^+^ T cells and contribute to better viral control.^47,48^ Therefore, the lack of significant improvement in T cell function in our study may be partially attributed to the absence of ATI. Fourthly, due to the lack of T Cell Receptor stimulation in suppressed individuals, these cells may not be adequately activated by even a slight rise in CA HIV RNA to effectively assess the impact of ICIs.^49^ Thus, optimizing the reversal of the HIV-1 latency may prove advantageous in enhancing specific T cell responses. The absence of significant differences observed in our study may also stem from our utilization of total and integrated HIV DNA to represent HIV viral reservoir, rather than intact HIV DNA. Additionally, T cell responses are not assessed immediately after each injection but rather 4 weeks later, which may be too delayed to detect any effect. Future studies with ICIs may incorporate higher doses, multiple dose administration, combination therapy with various ICIs, e.g., PD-1/PD-L1 inhibitors and CTLA-4 inhibitors or enhancers, recombinant human interleukin-15 (rhIL-15)^50,51^ and closer follow-up of T cell function to validate the potential to improve T cell function.

The incidence of AEs attributed to ASC22 and chidamide was low, with all being of low grade (CTCAE grade 1). These events primarily encompassed rash and subclinical hyperthyroidism. Importantly, none of the participants required pharmacological intervention, and no AEs necessitated discontinuation of the study treatment, which supports further clinical trials.

This study has several limitations. Firstly, baseline ART was consistently maintained throughout the study duration, with no assessments conducted for ATI. Secondly, the quantification of surrogate markers of the HIV reservoir relies on PCR-based techniques, which may lead to an overestimation of their magnitude as a significant proportion of the detected viral genomes are non-replicable. Due to limitations in sample size, we were unable to simultaneously measure replication-competent HIV and conduct Quantitative Viral Outgrowth Assays (QVOA), which would have contributed to a more comprehensive study.^52^ We also do not have enough samples to sort CD4^+^ T cells for HIV DNA determination, so we chose PBMCs for quantification. Thirdly, the enrollment for clinical trials exclusively included male participants. Therefore, the results may not be generalized to females. The study had limitations in terms of its single-arm design. Fourthly, Plasma drug concentrations were not monitored. Thus, the increased plasma viremia, elevated levels cell-associated unspliced HIV RNA, and decline in HIV DNA observed could potentially be attributed to naturally occurring longitudinal variation. However, this explanation seems unlikely, considering all participants had maintained stable suppression viremia for minimum of 2 years prior to enrollment.

The co-administration of ASC22 and chidamide demonstrates efficacy in potentiating HIV latency reversal; however, it falls short in effectively eradicating the HIV reservoir. Additionally, this combined approach significantly augments the ratios of CD4^+^ and CD8^+^ T_EM_ cells, it does not effectively enhance the function of HIV-specific CD8^+^ T cells. This strategy tends to have a more pronounced decrease in the viral reservoir of HIV in populations with stronger T cell functionality. The combination strategy shows the potential of effectively activate and reduce latent HIV reservoirs and highlights the role of enhanced HIV-specific T cell functionality in reducing viral reservoirs.

## Materials and methods

### Study design and participants

We performed an open-label, non-randomized, controlled study phase II clinical trial at the Shanghai Public Health Clinical Center, Shanghai, China. Our study enrolled adult PLWH who had received at least 24 months of ART and had two consecutive episodes of plasma HIV viral load less than 50 copies/mL, separated by a minimum of 12 months. All enrolled participants were receiving plasma HIV RNA testing at least once every six months or one year. Participants had baseline CD4^+^ T cell counts exceeding 250 cells/*μ*L and CD4/CD8 ratios <0.9. The main exclusion criteria included prior administration or exposure to ICIs, the presence of active autoimmune disease, and the occurrence of any severe acute illness within an eight-week timeframe. The clinical trial was approved by the Research Ethics Committee of the Shanghai Public Health Clinical Center (Ethics committee number: 2021-E040-01), and all participants signed written informed consent before being included in the study. The study has been registered on the ClinicalTrials.gov (NCT05129189).

### Procedures

Participants were administered ASC22 (1 mg/kg) via subcutaneous injection every 4 weeks for a total of 3 times. Chidamide (10 mg) was administered orally twice weekly for 12 weeks, while continuing ART (Fig. 1). ASC22 was supplied by Ascletis Pharma Inc., China, while chidamide was supported by Shenzhen Chipscreen Biosciences, China. The treatment duration of ASC22 combined with chidamide was 12 weeks, and the observation periods were 12 weeks after discontinuation of ASC22 and chidamide. Participants had a total of 11 visits during the study: once every 2 weeks during the treatment period, and once every 4 weeks during the observation period (Supplementary Table. 1). During the treatment period, samples were collected at weeks 0 (baseline), 4, 8, 12, and 24. ASC22 was administered at least 1 h after chidamide. Blood samples were collected each time before ASC22 administration. We evaluate the treatment effect of the previous cycle through each sampling period. Peripheral blood mononuclear cells (PBMCs) and plasma were immediately processed and cryopreserved for subsequent testing.

### Outcomes

The primary outcomes were the changes in total and integrated HIV DNA of PBMCs from baseline to week 24. The secondary endpoints included assessed by quantifying CA HIV RNA in PBMCs, and plasma HIV viral loads, as well as analyzing variations in the functionality of HIV Gag/Pol specific CD8^+^ T cells from baseline to post-treatments.^53^ Additionally, changes in CD4^+^ and CD8^+^ T cell counts, as well as CD4/CD8 ratios were monitored at weeks 0 (baseline), 4, 8, 12, and 24. Other outcome was determined by assessing the incidence and severity of adverse events (AEs), which were evaluated following the Adverse Event Common Terminology Criteria, version 6.0 (CTCAE V6.0). AEs were recorded from the initial dose of ASC22 through the safety follow-up period, extending to 24 weeks after the final dose. Solicited and unsolicited adverse reactions were registered from all volunteers. The causality of suspected adverse drug reactions was assessed using the WHO-Uppsala Monitoring Center (WHO-UMC) system.^54^ AEs were documented using Medical Dictionary for Regulatory Activities (MedDRA®; version 26.1) terms.

A greater than 2-fold increase in CA HIV RNA or plasma HIV viral loads exceeding the threshold of 50 copies/ml were considered indicative of HIV reservoir activation. If the fold change of total and integrated HIV DNA was less than 1, this was defined as a reduction in the HIV reservoir size. Improvement in T cell immune function was defined as a more than 2-fold rise in the proportions of IFN-γ or TNF-α expression by HIV Gag- or Pol-specific CD8^+^ T cells after HIV peptide pool stimulation.

### Quantification of CD4^+^ and CD8^+^ T cell counts and plasma HIV viral loads

Flow cytometry (BD FACS CantoTM, New Jersey) assessed CD4^+^ and CD8^+^ T cell counts, while plasma HIV viral loads were quantified via polymerase chain reaction (PCR) (Cobas X 480 and Cobas Z 480, Roche, Basel, Switzerland). All assays were performed at the clinical laboratory of the Shanghai Public Health Clinical Center.

### Quantitative detection of CA HIV RNA and total HIV DNA

Total cellular RNA and DNA were amplified and quantified from PBMCs using HIV DNA quantitative detection kit (SUPBIO, China) and CA HIV RNA quantitative detection kit (SUPBIO, China).^55^ The quantitative values for total HIV DNA (copy/*μ*L) and the quantitative numbers of cells (unit/*μ*L) were obtained, which were then used to calculate the copy number of total HIV DNA and CA HIV RNA per million PBMCs. The total HIV DNA assay displayed a lower detection limit of 20 copies/million PBMCs and a quantification range of 50-1 × 10^6^ copies/million PBMCs. For the CA HIV RNA, the lower detection limit was found to be 2 copies/reaction, while the linear range spanned from 2–2 × 10^5^ copies/reaction.

### A quantitative assay was conducted to measure HIV DNA integration

Integrated HIV DNA Quantitative was conducted using an integrated HIV DNA quantitative detection kit (SUPBIO, China), and the principle of nested fluorescent quantitative PCR was adopted. In the first round of PCR, upstream and downstream primers were designed on the HIV gene and the Alu fragment of the human genome, respectively. In the second round of fluorescent quantitative PCR, the first round of PCR products (2 *μ*L) were quantified with HIV-specific primers and probe detection. After obtaining the quantitative value of integrated HIV DNA (copies/*μ*L) and the quantitative numbers of cells (cells/*μ*L), the integrated HIV DNA copy number per million PBMCs was calculated. The integrated HIV DNA assay detected a lower limit of 10 copies/10^6^ PBMCs, and the quantitative range was 10–10^6^ copies/10^6^ PBMCs.

### Peptides

The CD8^+^ T cell epitope of the HIV-1 Gag and Pol protein were sourced from the HIV database (https://www.hiv.lanl.gov/mojo/immunology/search/ctl/form.html), which consists of 8 to 11 amino acids. A total of 32 CD8^+^ T cell epitopes were selected and submitted to GenScript Biotech Corp company for chemical synthesis. The peptides sequences were Gag (18–27) KIRLRPGGKK, Gag (45–53) AVNPGLLET, Gag (63–72) QLQPSLQTGS, Gag (77–86) SLYNTVATLY, Gag (87–95) CVHQRIEVK, Gag (148–158) SPRTLNAWVKV, Gag (193–203) GHQAAMQMLKE, Gag (214–224) RLHPVHAGPIA, Gag (240–251) TSTLQEQIGWMT, Gag (249–258) WMTNNPPIPV, Gag (267–274) ILGLNKIV, Gag (275–283) RMYSPTSIL, Gag (291–301) EPFRDYVDRFY, Gag (349–359) ACQGVGGPGHK, Gag (362–370) VLAEAMSQV, Gag (368–376) (SQVTNSATI) and Pol (101–110) KMIGGIGGFI, Pol (132–140) LVGPTPANI, Pol (158–167) SPIETVPVKL, Pol (188–196) ALVEICTEM, Pol (248–257) GIPHPAGLKK, Pol (263–273) VLDVGDAYFSV, Pol (282–290) YTAFTIPSI, Pol (313–321) AIFQSSMTK, Pol (336–344) YQYMDDLYV, Pol (464–473) ILKEPVHGVY, Pol (495–505) QIYQEPFKNLK, Pol (571–579) FVNTPPLVK, Pol (640–648) ALQDSGLEV, Pol (886–895) HLKTAVQMAV, Pol (894–903) AVFIHNFKRK, Pol (918–926) (IIATDIQTK), respectively. Each peptide, synthesized at a quantity of 4 mg, was dissolved in DMSO first, then diluted to 1 mg/ml in PBS, and stored at −80 °C in the end for future use.

### Flow cytometry to measure HIV-specific CD8^+^ T cells responses

PBMCs were resuspended into 96 U-well plates at 6 × 10^5^ cells/well, then stimulated with Gag and Pol peptide pool (5 μg/ml of each selected single peptide), and PMA/Ionomycin cocktail (eBioscience, USA) as a positive control. After a 2-h incubation at 37 °C, adding protein transport inhibitor cocktail (eBioscience, USA), and incubated for an additional 10 h. The stimulated cells were stained with various cell surface markers, including anti-human CD3- APC-H7 (Clone SK7, BD Pharmingen, USA), anti-Human CD4-FITC (Clone RPA-T4, BD Pharmingen, USA), anti-human CD8a-BV650 (Clone RPA-T8, Biolegend, USA), anti-Human CCR7-PE (Clone REA108, MACS, GER), anti-Human CD45RA-APC (Clone HI100, BD Pharmingen, USA), anti-human CD279 (PD-1)-BV785 (Clone EH12.2H7, Biolegend, USA), and LIVE/DEAD Aqua reagent (Invitrogen, USA). Subsequently, the stimulated cells were incubation in fixation and permeabilization solution (BD Biosciences, USA) half hours, then stained with intracellularly markers including anti-human TNF-α-PE-Cy7 (Clone MAb11, Invitrogen, USA) and anti-human IFN-γ-BV421 (Clone 4 S.B3, BD Horizon, USA). Stimulated PBMCs were analyzed using BD LSRFortessa flow cytometer, with 100,000 events recorded for each sample (Supplementary Fig. 8).

### Statistical analysis

We employed repeated measures ANOVA to evaluate whether there were significant longitudinal changes in numerical outcome measures compared to baseline. Paired t-tests, Mann-Whitney U or Wilcoxon signed-rank tests were employed to evaluate changes from baseline to specific time points, with the choice of test depending on the data distribution. For post hoc analyses exploring correlations, we employed Spearman’s or Pearson’s rank correlation, depending on the nature of the data. Statistical analyses were conducted using SPSS 15.0 statistical software (Chicago, IL, USA) and GraphPad Prism 8.0 (San Diego, CA, USA). *P* value < 0.05 was deemed statistically significant.

## Supplementary information

Supplementary_Materials

Study protocol

## Data Availability

The dataset produced during this study is available upon request from the corresponding author (qtchenjun@163.com).
