# Supplementary material for: Anti-PD-L1 antibody ASC22 in combination with a histone deacetylase inhibitor chidamide as a “shock and kill” strategy for ART-free virological control: a phase II single-arm study
Source: Signal Transduct Target Ther. 2024 Sep 9;9:231. doi: 10.1038/s41392-024-01943-9 (PMC11381521; doi:10.1038/s41392-024-01943-9)
Supplement: Supplementary file 1 — Supplementary_Materials [file 41392_2024_1943_MOESM1_ESM.doc]

Supplementary Materials for

**Anti-PD-L1 antibody ASC22 in combination with chidamide as a “shock and kill” strategy for ART-free virological control: a phase Ⅱ single-arm study**

Luling Wu, ZhihangZheng, Jingna Xun, Li Liu, Jiangrong Wang, Xinyu Zhang, Yueming Shao, Yinzhong Shen, Renfang Zhang, MinZhang, Meiyan Sun, Tangkai Qi, Zhenyan Wang, Shuibao Xu, Wei Song, Yang Tang, Bihe Zhao, Zichen Song, Jean-Pierre Routy, Hongzhou Lu, Jun Chen

Correspondences to: Jun Chen (qtchenjun@163.com);

Hongzhou Lu (luhongzhou@fudan.edu.cn)

**This PDF file includes:**

Figures. S1 to S8

Tables S1


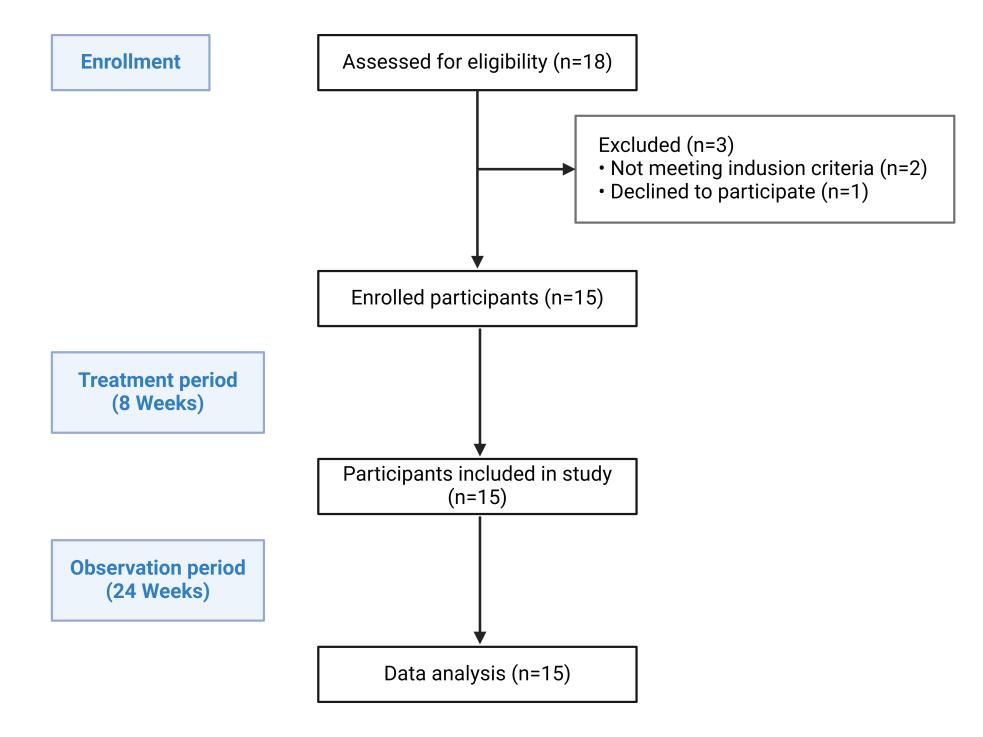


**Figure. S1. CONSORT flowchart for the present study.**

**Figure. S2. Individual dynamics of fold changes in cell-associated (CA) HIV RNA (a), total HIV DNA (b), and integrated HIV DNA (c) at weeks 0 (baseline), 4, 8, 12, and 24.** The black dotted line means total HIV DNA and the integrated HIV DNA less than two- fold change, and the gray shade represents less than one- fold change.


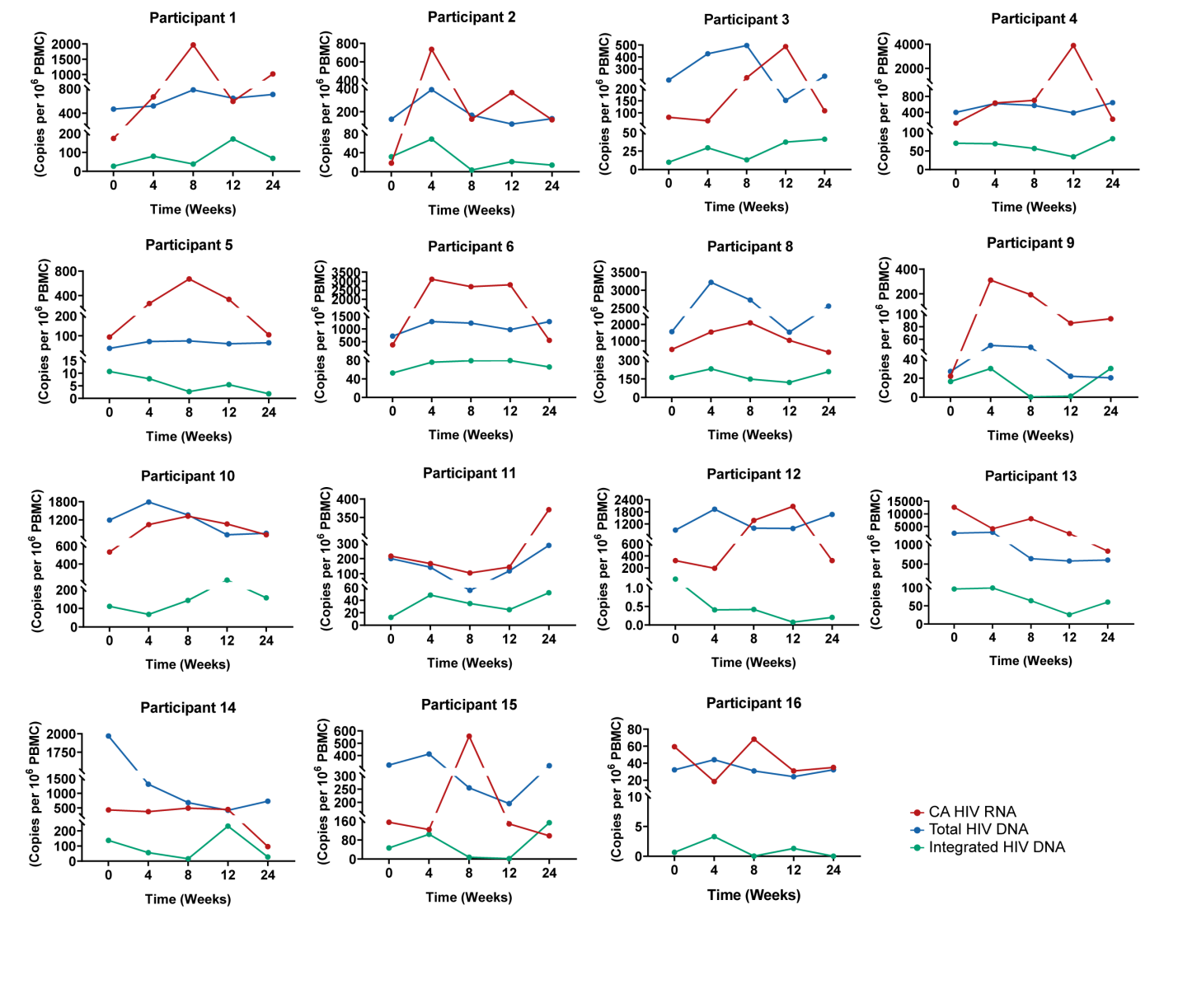


**Figure. S3. Changes in CA HIV RNA, total HIV DNA and** **integrated HIV DNA in each participants at weeks 0 (baseline), 4, 8, 12, and 24.**

**Figure. S4. Changes in % of T_EM_ (a), T_N_ (b), T_CM_ (c), and T_EMRA_ (d) subsets of CD4^+^ T (left) and CD8^+^ T cells (right) at weeks 0 (baseline), 4, 8, 12, and 24.** ns means not significant.

Abbreviations: T_CM_, Central Memory T cell ; T_CM_, Central Memory T cell; T_EMRA_, Terminally Differentiated Effector Memory T cell; T_N_, Naïve T cell.


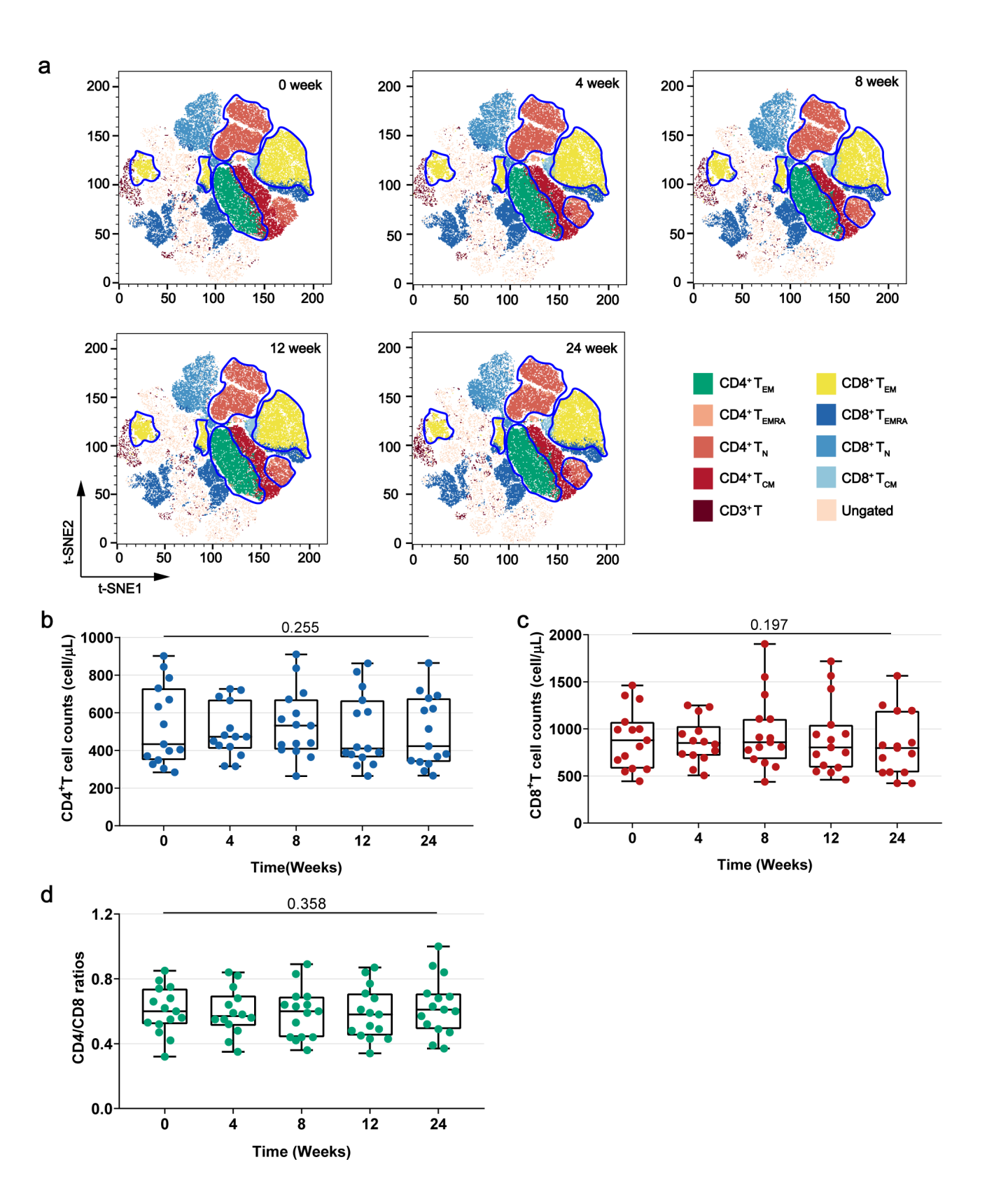


**Figure. S5. Effects of combination therapy with ASC22 and chidamide on CD4^+^ and CD8^+^ T cells proliferation. (a) T-SNE plot of dynamics of T cell phenotype throughout treatment and observation periods. Changes in CD4^+^T cell counts (b), CD8^+^T cell counts (c), and CD4/CD8 ratios (d) at weeks 0 (baseline), 4, 8, 12, and 24.**

Abbreviations: T_CM_, Central Memory T cell; T_EM_, Effector Memory T cell; T_EMRA_, Terminally Differentiated Effector Memory T cell; T_N_, Naïve T Cell.


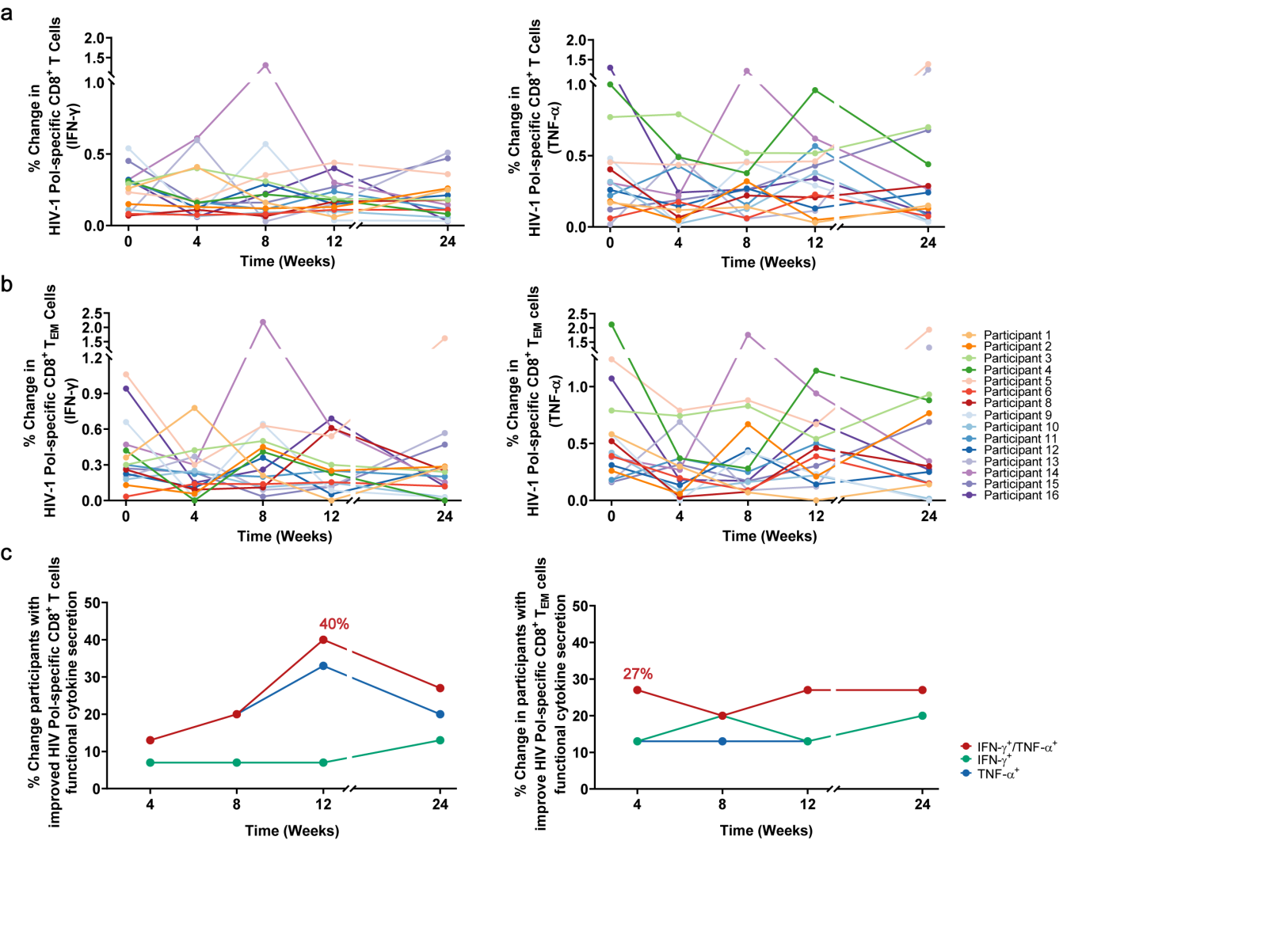


**Figure. S6. Changes in HIV Pol-specific CD8^+^ T cell responses. (a) Dynamics of HIV Pol-specific CD8^+^ T cells responses in each participant at weeks 0 (baseline), 4, 8, 12, and 24, represented for IFN-γ (left) and TNF-α (right). (b) Changes in the dynamics of HIV Pol-specific CD8^+^ T_EM_ cells responses at weeks 0 (baseline), 4, 8, 12, and 24 are presented for each participant, with functional cytokines IFN-γ (left) and TNF-α (right) shown.** **(c) Changes in the proportion of participants with an improvement in HIV Pol-specific CD8^+^ T cells (left) and CD8^+^ T_EM_ cells (right) secreting functional cytokines from baseline to week 24.** Improvement in T cell immune function was defined as a more than 2-fold increase in the expression of IFN-γ or TNF-α in HIV Pol- specific CD8^+^ T cells.


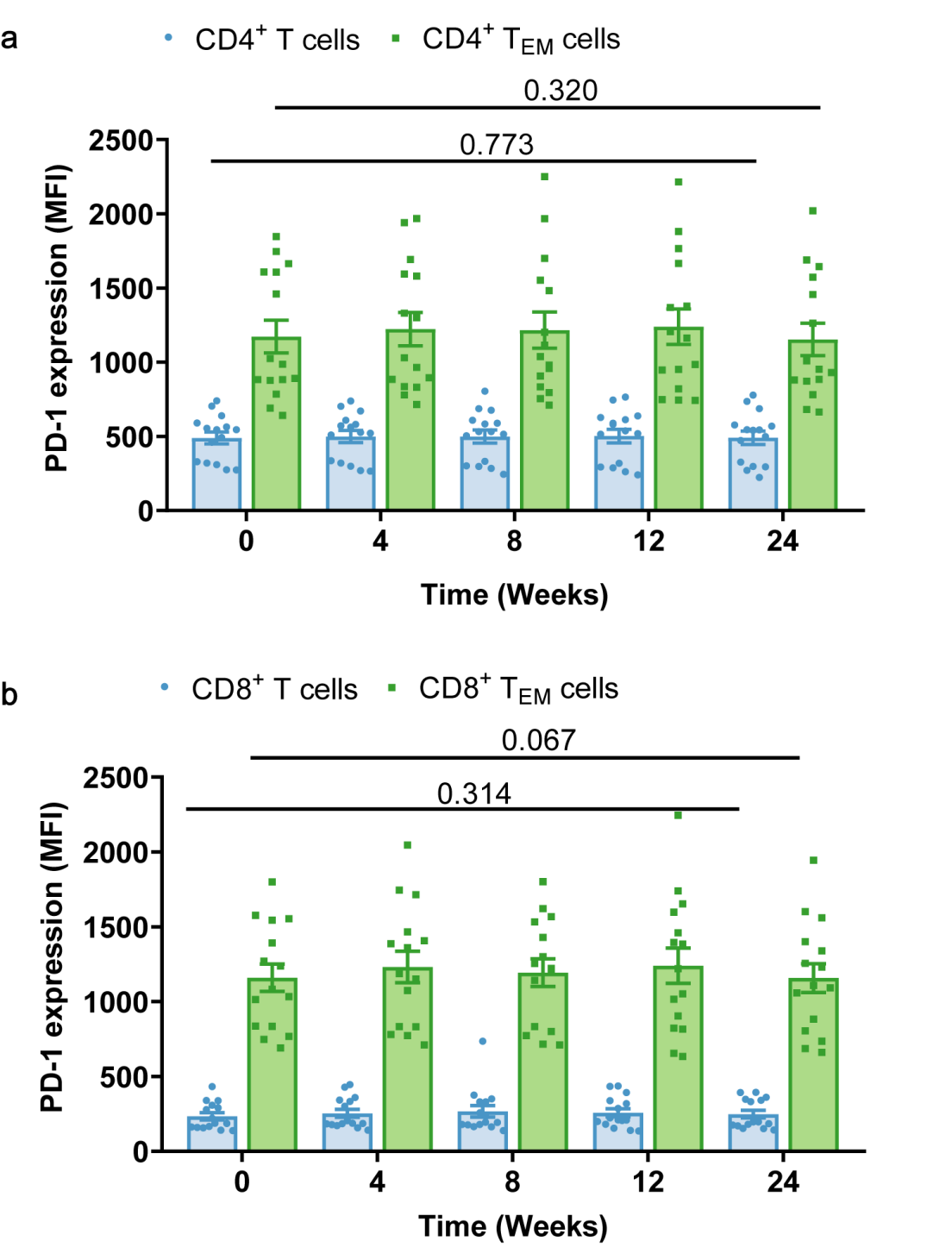


**Figure. S7. Changes in the fluorescence expression of PD-1 in CD4^+^ and CD8^+^ T cells (a), as well as T_EM_ cells (b), across different treatment and observation cycles.**

Abbreviations: MFI, Mean Fluorescence Intensity; T_EM_, Effector Memory T cell.


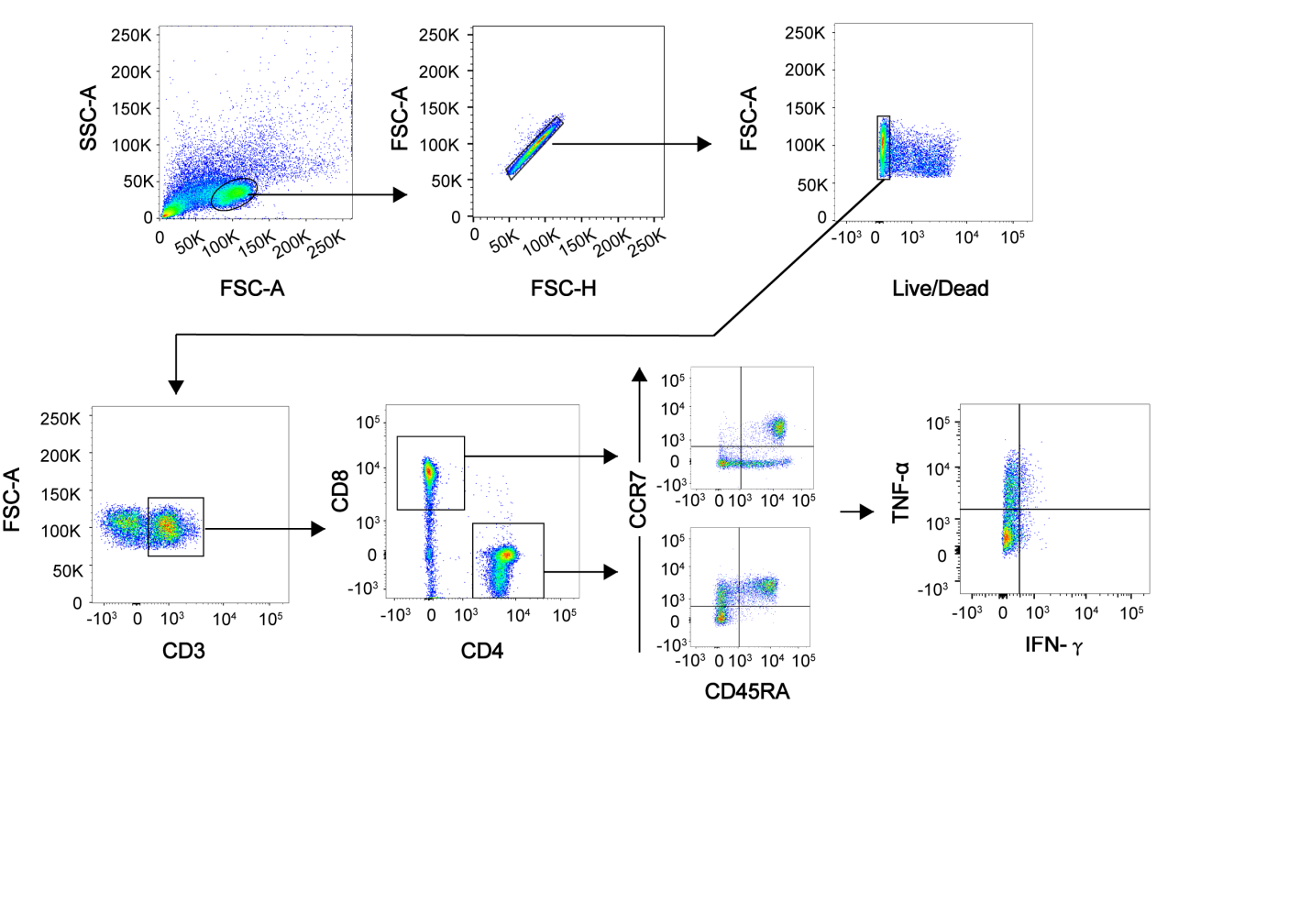


**Figure. S8. Flow cytometry gating strategy.** T cell subsets were defined as follows: naïve (T_N_, CD45RA^+^ CCR7^+^), central memory (T_CM_, CD45RA^-^ CCR7^+^), effector memory (T_EM_, CD45RA^-^ CCR7^-^), and terminal differentiated memory (T_EMRA_, CD45RA^+^ CCR7^-^).

Abbreviations: CCR7, Chemokine Receptor 7.

**Table S1. Clinical trial procedures**

|  | **Screening period^a^** | | **Treatment period** | | | | | | **Screening period** | | |
| --- | --- | --- | --- | --- | --- | --- | --- | --- | --- | --- | --- |
|  | **V1** | **V2** | **V3** | **V4** | **V5** | **V6** | **V7** | **V8** | **V9** | **V10** | **V11** |
|  | **Screening** | **Baseline** | **Week 2** | **Week 4** | **Week 6** | **Week 8** | **Week 10** | **Week 12** | **Week 16** | **Week 20** | **Week 24** |
| **Visit time** | **Day- 28~Day-2** | **Day 0** | **Day15**  **±2** | **Day 29**  **±2** | **Day 43**  **±2** | **Day 57**  **±2** | **Day 71**  **±2** | **Day 85±2 /Early termination** | **Day 113±5** | **Day 141±5** | **Day 169±2** |
| **Informed consent** | **×** |  |  |  |  |  |  |  |  |  |  |
| **Demographic** | **×** |  |  |  |  |  |  |  |  |  |  |
| **Medical history, treatment history, and history of drug abuse** | **×** |  |  |  |  |  |  |  |  |  |  |
| **Physical examination** | **×** | **×** | **×** | **×** | **×** | **×** | **×** | **×** | **×** | **×** | **×** |
| **Blood pregnancy test**  **(if applicable)** | **×** |  |  | **×** |  | **×** |  | **×** |  |  |  |
| **Height, weight, vital signs^b^** | **×** | **×** | **×** | **×** | **×** | **×** | **×** | **×** | **×** | **×** | **×** |
| **Blood routine^d^** | **×** | **×^a^** | **×** | **×** | **×** | **×** | **×** | **×** | **×** | **×** | **×** |
| **Blood biochemistry**  **(including myocardial enzymes)^e^** | **×** | **×^a^** | **×** | **×** | **×** | **×** | **×** | **×** | **×** | **×** | **×** |
| **Urine routine^f^** | **×** | **×^a^** | **×** | **×** | **×** | **×** | **×** | **×** | **×** | **×** | **×** |
| **Coagulation function^g^** | **×** | **×^a^** | **×** | **×** | **×** | **×** | **×** | **×** | **×** | **×** | **×** |
| **Thyroid function^h^** | **×** | **×^a^** |  | **×** |  | **×** |  | **×** |  |  | **×** |
| **Adrenaline^i^** | **×** | **×^a^** |  | **×** |  | **×** |  | **×** |  |  |  |
| **Blood sugar^j^** | **×** | **×^a^** |  | **×** |  | **×** |  | **×** |  |  |  |
| **Twelve-lead electrocardiogram^c^** | **×** | **×** | **×** | **×** | **×** | **×** | **×** | **×** |  |  |  |
| **Chest CT scan** | **×** |  |  |  | **×** |  |  | **×** |  |  |  |
| **Cardiac ultrasound** | **×** |  |  |  | **×** |  |  | **×** |  |  |  |
| **Abdominal ultrasonography (B ultrasound)** | **×** |  |  |  | **×** |  |  | **×** |  |  |  |
| **Plasma HIV Viral load (HIV-1 RNA)** | **×** | |  | **×** |  | **×** |  | **×** |  |  |  |
| **T cell immune^k^** | **×** | |  | **×** |  | **×** |  | **×** |  |  |  |
| **HIV Gag-specific CD8^+^T** | **×** | |  | **×** |  | **×** |  | **×** |  |  |  |
| **HIV reservoir**  **(HIV-1 DNA)** | **×** | |  | **×** |  | **×** |  | **×** |  |  |  |
| **ASC22 administration** |  | **×** |  | **×** |  | **×** |  |  |  |  |  |
| **Chidamide dispensing drug** |  | **×** | **×** | **×** | **×** | **×** | **×** |  |  |  |  |
| **Distribute medication diary cards** |  | **×** | **×** | **×** | **×** | **×** | **×** |  |  |  |  |
| **Recycling medication diary cards** |  | **×** | **×** | **×** | **×** | **×** | **×** | **×** |  |  |  |
| **AEs** | **×** | | | | | | | | **×** | **×** | **×** |
| **Combination therapy** | **×** | | | | | | | | **×** | **×** | **×** |
| **Summary and analysis of test completion status** |  |  |  |  |  |  |  |  | **×** | | |

a. When the screening test and the day of drug administration coincide, relevant laboratory tests can be shared. Typically, results of pertinent safety tests conducted within approximately one week from the date of signing the informed consent form to the day of drug administration are acceptable.

b. Height measurement is only conducted during the screening period. Vital signs include body temperature, heart rate, respiration rate, and blood pressure.

c. ECG should be performed before ASC22 administration (within ±30 minutes) on the day of drug administration.

d. Blood routine tests include total WBC, WBC differential count (neutrophil [N] absolute count, lymphocyte [L] count, monocyte [M] count, eosinophils [E] count, absolute basophil [B] count), total RBC, HB, PLT, and HCT.

e. Blood biochemistry tests include total protein, albumin, total cholesterol, LDL, HDL, triglycerides, urea nitrogen/urea, uric acid, creatinine, alkaline phosphatase, lactate dehydrogenase, total bilirubin, direct bilirubin, indirect bilirubin, AST, ALT, calcium, phosphorus, magnesium, potassium, sodium, chloride, and serum amylase. Cardiac enzyme spectrum examination includes troponin-I, CK, and CK-MB.

f. Urine routine includes specific gravity, pH, urine sugar, protein, casts, ketone bodies, blood cells, and RBC.

g. Coagulation function includes PT, APTT, INR, FIB, TT, and D-dimer.

h. Thyroid function includes TSH, FT3, FT4, T3, T4. No relevant drug interference exists, and the test results within two weeks of the screening or baseline period are acceptable.

i. Adrenaline testing includes ACTH and cortisol. There is no interference from related drugs, and the test results are acceptable within two weeks of the screening or baseline period.

j. Blood glucose examination includes blood glucose and glycated hemoglobin. The glycated hemoglobin test results within 12 weeks during the screening and baseline periods are acceptable.

k. T-cell immune includes CD3^+^T cell count, CD4^+^T cell count, and CD8^+^T cell count.
