# Supplementary material for: Anti-PD-L1 antibody ASC22 in combination with a histone deacetylase inhibitor chidamide as a “shock and kill” strategy for ART-free virological control: a phase II single-arm study
Source: Signal Transduct Target Ther. 2024 Sep 9;9:231. doi: 10.1038/s41392-024-01943-9 (PMC11381521; doi:10.1038/s41392-024-01943-9)
Supplement: Supplementary file 2 — Study protocol [file 41392_2024_1943_MOESM2_ESM.doc]

**Clinical Trial Protocol**

| **Title** | Anti-PD-L1 antibody ASC22 in combination with chidamide as a “shock and kill” strategy for HIV functional cure: a phase Ⅱ study |
| --- | --- |
| **Sponsor** | Shanghai Public Health Clinical Center, Fudan University, Shanghai, China |
| **Protocol Number** | V20220703 |
| **Version** | V1.3 |
| **Date** | June 3^th^, 2022 |

**Confidentiality statement**

The information in this test protocol is confidential, and any information added to this protocol belongs to the unit. The information provided in this trial protocol is intended to fund an upcoming clinical trial and is provided confidentially to investigators participating in this clinical trial. The researcher may disclose the contents of the protocol to other participants in the clinical trial or to the managers and ethics committees of the clinical trial institution where the researcher is located for the same purpose, subject to the following conditions: All information will only be used when authorized to carry out this protocol. For use in the clinical studies described, the contents of this protocol cannot be used in other clinical trials. Except for the relevant information that needs to be introduced to the subjects when obtaining informed consent, data from this protocol can only be disclosed to this individual or group with prior written permission.

**Table of Contents**

Confidentiality statement..............................................................................................1

List of Abbreviations.....................................................................................................4

Program summary.........................................................................................................6

ASC22 clinical trial flow chart....................................................................................10

1 Background...............................................................................................................13

1.1Immune Escape Of Virus........................................................................................13

1.2 Introduction To Trial Drugs...................................................................................14

1.3 Preclinical Studies.................................................................................................14

1.3.1 Pharmacokinetics...............................................................................................14

1.3.2 Toxicology..........................................................................................................15

1.3.3 Pharmacodynamics.............................................................................................16

1.3.3.1 Anti-HIV Infection..........................................................................................16

1.3.3.2 Anti-HBV Infection.........................................................................................16

2 Study Purpose...........................................................................................................17

3 Study Design............................................................................................................17

3.1 Basis For Dose Selection......................................................................................17

3.1.1 Phase I Pharmacokinetic Study..........................................................................17

3.1.2 Effectiveness Research......................................................................................18

3.1.2.1 Anti-HBV Efficacy.........................................................................................18

3.1.2.2 Anti-tumor Efficacy........................................................................................19

3.1.3 Security..............................................................................................................20

3.1.4 The Steady-state Target Concentration Of ASC22............................................20

3.2 Inclusion And Exclusion Criteria..........................................................................21

3.2.1 Inclusion Criteria................................................................................................21

3.2.2 Exclusion criteria................................................................................................21

3.2.3 Exit Criteria........................................................................................................22

3.2.3.1 Withdrawal At The Investigator's Discretion...................................................22

3.2.4 Post-Exit Procedures...........................................................................................23

3.2.5 Criteria for Terminating the Study......................................................................23

4 Drugs.........................................................................................................................24

4.1 Investigational drugs..............................................................................................24

4.2 Antiviral regimen drugs..........................................................................................24

5 Clinical trial content..................................................................................................24

5.1 Sample size............................................................................................................24

5.2 Test Process............................................................................................................24

5.3 Flowchart Of The Study........................................................................................24

5.4 Test Result..............................................................................................................25

5.4.1 Security Monitoring............................................................................................25

5.4.2 The Activation of HIV Reservoir Detection......................................................25

5.4.3 The changes of HIV reservoir.............................................................................25

5.4.4 Immunological Examination...............................................................................26

5.4.5 Laboratory Examination and Organ Function Evaluation..................................26

6 Efficacy and safety outcomes....................................................................................26

7 Research Monitoring, Auditing, and Inspection........................................................26

7.1 Research monitoring..............................................................................................26

7.2 Research Auditing and Inspection..........................................................................27

8 Publication of Research Data....................................................................................27

9 Investigator Qualifications........................................................................................27

10 Ethical and Legal Issues..........................................................................................27

10.1 EC.........................................................................................................................27

10.2 Informed Consent.................................................................................................28

10.3 Confidentiality......................................................................................................28

11 Data storage.............................................................................................................28

12 References...............................................................................................................28

**List of Abbreviations**

| ACTH | Adrenocorticotropic hormone |
| --- | --- |
| ADCC | Antibody-dependent cytotoxicity |
| AEs | Adverse events |
| APTT | Activated partial thromboplastin time |
| ANC | Absolute neutrophil count |
| BIRC | Blinded independ review committee |
| BMI | Body mass index |
| CA HIV RNA | cell-associated HIV RNA |
| CDC | Complement-dependent cytotoxicity |
| CHB | Chronic hepatitis B |
| CK | Creatine kinase |
| CK-MB | Creatine kinase isoenzyme |
| CRC | Colorectal cancer |
| CRF | Case report form |
| CTCAE | Common Terminology Criteria for Adverse Events |
| CTLA-4 | Cytotoxic T lymphocyte-associated antigen 4 |
| Cys | Cysteine residue |
| dAb | Domain antibody |
| DC | Dendritic cell |
| DLT | Dose-limiting toxicity |
| DOR | Duration of response |
| dMMR | DNA mismatch repair deficienc |
| EC | Ethical Committee |
| ECG | Electrocardiogram |
| FIB | Fibrinogen |
| FT3 | Free triiodothyronine |
| FT4 | Free tetraiodothyronine |
| GC | Gastric cancer |
| GCP | Good Clinical Practice |
| HB | Hemoglobin |
| HBsAg | Hepatitis B surface antigen |
| HCT | Hematocrit |
| HDACI | Histone deacetylase inhibitor |
| HDL | High-density lipoprotein |
| HIV | Human immunodeficiency virus |
| IgG | Immunoglobin G |
| IEC | Independent ethics committeee |
| INR | International normalized ratio |
| L | Lymphocyte |
| LAG-3 | Lymphocyte activating gene-3 |
| ICIs | Immune checkpoint inhibitors |
| LCMV | Lymphocytic choriomeningitis virus |
| LDL | Low-density lipoprotein |
| MSI-H | Microsatellite unstable |
| N | Neutrophil |
| NDA | New drug application |
| NOAEL | No observed adverse effect level |
| ORR | Objective response rate |
| OS | Overall survival |
| PBMCs | Peripheral blood mononuclear cells |
| PD | Progressive disease |
| PD-L1 | Anti-programmed cell death protein 1 |
| PFS | Progression-free survival |
| PLT | Platelets |
| PLWH | People living with HIV |
| PRs | Partial responses |
| PT | Prothrombin time |
| RBC | Red blood cell |
| SD | Stable disease |
| Ser | Serine residue |
| SGOT/AST | Serum aspartate aminotransferase |
| SGPT/ALT | Serum alanine transaminase |
| SIV | Simian immunodeficiency virus |
| T3 | Triiodothyronine |
| T4 | Tetraiodothyronine |
| TdP | Torsades de pointes |
| TEAEs | Treatment-emergent adverse events |
| TIM-3 | T cell immunoglobulin 3 |
| TSH | Thyroid stimulating hormone |
| TT | Thrombin time |
| ULN | Upper limit of normal |
| US | United States |
| V_H_H | Variable domain of the heavy-chain of heavy-chain antibody |
| WBC | White blood cell count |
| WHV | Woodchuck hepatitis virus |

**Program summary**

| Basic information | Protocol Number: V20220703  Version/Date: V1.3/20220703  Study drug: Recombinant humanized PD-L1 single domain antibody Fc fusion protein injection  Drug code: ASC22 (previously used code KN035, Envafolimab)  Drug specifications: 1 ml: 200 mg (1 ml/ampoule)  Drug production unit: Alphamab Oncology, jiangsu, China |
| --- | --- |
| Title | Anti-PD-L1 antibody ASC22 in combination with chidamide as a “shock and kill” strategy for HIV functional cure: a phase Ⅱ study |
| Study purposes | Assessing whether ASC22 in combination with chidamide can reduce HIV reservoirs in PLWH with virologically suppressed |
| Study design | Single center, prospective, single-arm, phase Ⅱ study |
| Population | PLWH |
| Sample size | This study estimates that 12 participants will complete the trial, and the dropout rate will be controlled within 15%, so 15 participants are planned to be enrolled. |
| Study treatment | Participants received a subcutaneous injection of ASC22 (1mg/kg) once every 4 weeks for a total of 3 times. Chidamide (10 mg) was administered orally twice weekly for 12 weeks, while continue ART |
| Inclusion criteria | (1) Individuals diagnosed with HIV infection;  (2) Age ≥ 18 years old;  (3) BMI ≥ 18.0 to < 35.0 kg/m^2^;  (4) Capable and willing to comply with the time requirements for study visits and assessments;  (5) Currently receiving ART for at least 24 months, with two consecutive plasma HIV-1 RNA levels <50 copies/ml, separated by an interval of at least 12 months;  (6) During the screening period, CD4^+^ T cell count is ≥ 250 cells/µl (including the boundary value), and CD4/CD8 ratio is < 0.9;  (7) Agree to use contraception during participation in the project and for up to 6 months after the end of the trial;  (8) Willing to provide informed consent. |
| Exclusion criteria | (1) Those who have suffered from any serious acute illness within 8 weeks;  (2) Subjects with a history of active autoimmune disease or autoimmune disease requiring systemic treatment;  (3) Prior treatment/exposure to any other ICIs (e.g., PD-1, anti-PD-L1, anti-PD-L2, anti-CTLA4, etc.);  (4) Patients who have undergone the following treatments:  a) Received other anti-latent drug treatments within 30 days before enrollment;  b) Underwent radiotherapy or chemotherapy within 30 days before screening;  c) Received immunosuppressive therapy within 60 days before screening;  d) Received immunomodulatory agents (e.g., interleukins, interferons), hydroxyurea, or foscarnet within 60 days before screening;  e) Received an HIV vaccine or systemic cytotoxic chemotherapy within 60 days before screening;  f) Previously received IgG treatment;  g) Underwent blood transfusion or cell growth factor treatment within 90 days before screening;  h) Currently using or planning to use rifampicin, rifabutin, and other drugs during screening;  (5) Individuals whose laboratory examinations meet the following criteria:  a) ANC < 1.50× 10^9^/L; Hb < 105 g/L (for men) or < 95 g/L (for women); platelets < 75× 10^9^/μL; INR > 1× ULN;  b) SGPT/ALT > 1.5× ULN, SGOT/AST > 1.5× ULN, total bilirubin, direct bilirubin > 1.5× ULN, serum creatinine > 1.5× ULN, and the abnormalities are clinically significant;  c) Clinical significance of five thyroid function abnormalities, including T3, T4, FT3, FT4, and TSH;  d) Abnormal epinephrine test with clinical significance, including at least ACTH and cortisol;  e) Abnormal blood glucose and glycosylated hemoglobin tests with clinical significance;  (6) Abnormal and clinically significant findings on the twelve-lead electrocardiogram at the time of enrollment;  (7) Presence of interstitial changes on chest CT examination at the time of enrollment;  (8) Individuals with severe heart disease, symptomatic or asymptomatic arrhythmia;  (9) Patients co-infected with HBV, HCV, syphilis, other liver diseases, and diabetic patients;  (10) Individuals with active or suspected malignant tumors or a history of malignant tumors within the past five years (except for basal cell skin or cervical cancer in situ);  (11) Individuals with a history of tuberculosis or active tuberculosis;  (12) Subjects with psychiatric or substance abuse disorders known to interfere with their ability to meet study requirements;  (13) Individuals who have received immunomodulatory or immunosuppressive treatments (including any dose of IV/oral [PO] steroids, but excluding inhalation, topical, or local injection steroids) within 24 weeks before the first dose of the study drug;  (14) Pregnant and lactating women and individuals, both men and women, who plan to have children during the study;  (15) Individuals with mental health conditions or substance abuse issues that may interfere with the test;  (16) Patients who have used HDACI, such as valproic acid, butyrate, phenylbutyrate, etc., may be enrolled after a 28-day washout;  (17) Patients with severe cardiac insufficiency (New York Heart Association [NYHA] cardiac insufficiency class IV);  (18) Patients with a history of arterial thromboembolic events, including myocardial infarction, unstable angina; cerebrovascular accident, or transient ischemic attack, within six months before enrollment in treatment; standard treatment of uncontrolled hypertension (systolic blood pressure ≥ 150 mmHg or diastolic blood pressure ≥ 100 mmHg); and patients with cardiomyopathy;  (19) Patients with a significant QT/QTC interval prolongation during the screening period (e.g., repeated measurements show QTc interval > 450 milliseconds, or other conditions that may lead to TdP risk, such as heart failure; hypokalemia, familial long QT syndrome) or concurrent use of drugs that may prolong the QT/QTc interval;  (20) Individuals with known allergies or anti-drug antibodies to the drugs or excipients used in this trial;  (21) Individuals deemed unsuitable to participate in this trial by the researcher. |
| Primary outcomes | The primary outcomes were assessed by quantifying changes in cell-associated (CA) HIV RNA, total HIV DNA, integrated HIV DNA in PBMCs, and plasma HIV RNA, as well as by analyzing variations in the functionality of HIV Gag- and Pol- specific CD8^+^ T cells from baseline to post-treatments. In addition, changes in CD4^+^ T cell counts, CD8^+^ T cell counts, and CD4/CD8 ratios were monitored at baseline and at different time points during treatment. |
| Secondary endpoints | The related symptoms and laboratory tests need to be monitored. These symptoms may include vomiting, fatigue, rash, itching, diarrhea, constipation, borborygmi, fever/chills, headache, palpitations, heat sensation, changes in appetite, gastrointestinal pain, gastroesophageal reflux, touch sensitivity, taste and smell disturbances, dizziness, muscle or joint pain, blisters, oral lesions, conjunctivitis, facial edema, and more.  The laboratory tests include blood routine, liver and kidney function, urine routine, myocardial enzymes, blood sugar, blood coagulation, electrolytes, electrocardiogram, chest CT examination, cardiac ultrasound examination, and abdominal B-ultrasound for liver and kidney examination.  The severity of AEs will be graded according to the CTCAE version 6.0. AEs are defined as any adverse medical events occurring in patients or clinical research participants taking a drug product, regardless of causality with the treatment. The investigator will assess the relationship of each event to the study drug as either reasonably likely or not reasonably likely. AEs are divided into grades 1-5, with grade 3 being serious but not life-threatening and requiring hospitalization, grade 4 being life-threatening and requiring hospitalization, and grade 5 indicating an AE-related death requiring immediate intervention. |
| Statistical Analysis | Statistical analyses were carried out using GraphPad Prism 8.0 (San Diego, CA, USA) and SPSS 15.0 statistical software (Chicago, IL, USA). P-value (P) below 0.05 was considered statistically significant. |

**Table 1 ASC22 clinical trial flow chart**

|  | **Screening period^a^** | | **Treatment period** | | | | | | **Screening period** | | |
| --- | --- | --- | --- | --- | --- | --- | --- | --- | --- | --- | --- |
|  | **V1** | **V2** | **V3** | **V4** | **V5** | **V6** | **V7** | **V8** | **V9** | **V10** | **V11** |
|  | **Screening** | **Baseline** | **Week 2** | **Week 4** | **Week 6** | **Week 8** | **Week 10** | **Week 12** | **Week 16** | **Week 20** | **Week 24** |
| **Visit time** | **Day- 28~Day-2** | **Day 0** | **Day15**  **±2** | **Day 29**  **±2** | **Day 43**  **±2** | **Day 57**  **±2** | **Day 71**  **±2** | **Day 85±2 /Early termination** | **Day 113±5** | **Day 141±5** | **Day 169±2** |
| **Informed consent** | **×** |  |  |  |  |  |  |  |  |  |  |
| **Demographic** | **×** |  |  |  |  |  |  |  |  |  |  |
| **Medical history, treatment history, and history of drug abuse** | **×** |  |  |  |  |  |  |  |  |  |  |
| **Physical examination** | **×** | **×** | **×** | **×** | **×** | **×** | **×** | **×** | **×** | **×** | **×** |
| **Blood pregnancy test**  **(if applicable)** | **×** |  |  | **×** |  | **×** |  | **×** |  |  |  |
| **Height, weight, vital signs^b^** | **×** | **×** | **×** | **×** | **×** | **×** | **×** | **×** | **×** | **×** | **×** |
| **Blood routine^d^** | **×** | **×^a^** | **×** | **×** | **×** | **×** | **×** | **×** | **×** | **×** | **×** |
| **Blood biochemistry**  **(including myocardial enzymes)^e^** | **×** | **×^a^** | **×** | **×** | **×** | **×** | **×** | **×** | **×** | **×** | **×** |
| **Urine routine^f^** | **×** | **×^a^** | **×** | **×** | **×** | **×** | **×** | **×** | **×** | **×** | **×** |
| **Coagulation function^g^** | **×** | **×^a^** | **×** | **×** | **×** | **×** | **×** | **×** | **×** | **×** | **×** |
| **Thyroid function^h^** | **×** | **×^a^** |  | **×** |  | **×** |  | **×** |  |  | **×** |
| **Adrenaline^i^** | **×** | **×^a^** |  | **×** |  | **×** |  | **×** |  |  |  |
| **Blood sugar^j^** | **×** | **×^a^** |  | **×** |  | **×** |  | **×** |  |  |  |
| **Twelve-lead electrocardiogram^c^** | **×** | **×** | **×** | **×** | **×** | **×** | **×** | **×** |  |  |  |
| **Chest CT scan** | **×** |  |  |  | **×** |  |  | **×** |  |  |  |
| **Cardiac ultrasound** | **×** |  |  |  | **×** |  |  | **×** |  |  |  |
| **Abdominal ultrasonography (B ultrasound)** | **×** |  |  |  | **×** |  |  | **×** |  |  |  |
| **Plasma HIV Viral load (HIV-1 RNA)** | **×** | |  | **×** |  | **×** |  | **×** |  |  |  |
| **T cell immune^k^** | **×** | |  | **×** |  | **×** |  | **×** |  |  |  |
| **HIV Gag-specific CD8^+^T** | **×** | |  | **×** |  | **×** |  | **×** |  |  |  |
| **HIV reservoir**  **(HIV-1 DNA)** | **×** | |  | **×** |  | **×** |  | **×** |  |  |  |
| **ASC22 administration** |  | **×** |  | **×** |  | **×** |  |  |  |  |  |
| **Chidamide dispensing drug** |  | **×** | **×** | **×** | **×** | **×** | **×** |  |  |  |  |
| **Distribute medication diary cards** |  | **×** | **×** | **×** | **×** | **×** | **×** |  |  |  |  |
| **Recycling medication diary cards** |  | **×** | **×** | **×** | **×** | **×** | **×** | **×** |  |  |  |
| **AE_S_** | **×** | | | | | | | | **×** | **×** | **×** |
| **Combination therapy** | **×** | | | | | | | | **×** | **×** | **×** |
| **Summary and analysis of test completion status** |  |  |  |  |  |  |  |  | **×** | | |

a. When the screening test and the day of drug administration coincide, relevant laboratory tests can be shared. Typically, results of pertinent safety tests conducted within approximately one week from the date of signing the informed consent form to the day of drug administration are acceptable.

b. Height measurement is only conducted during the screening period. Vital signs include body temperature, heart rate, respiration rate, and blood pressure.

c. ECG should be performed before ASC22 administration (within ±30 minutes) on the day of drug administration.

d. Blood routine tests include total WBC, WBC differential count (neutrophil [N] absolute count, lymphocyte [L] count, monocyte [M] count, eosinophils [E] count, absolute basophil [B] count), total RBC, HB, PLT, and HCT.

e. Blood biochemistry tests include total protein, albumin, total cholesterol, LDL, HDL, triglycerides, urea nitrogen/urea, uric acid, creatinine, alkaline phosphatase, lactate dehydrogenase, total bilirubin, direct bilirubin, indirect bilirubin, AST, ALT, calcium, phosphorus, magnesium, potassium, sodium, chloride, and serum amylase. Cardiac enzyme spectrum examination includes troponin-I, CK, and CK-MB.

f. Urine routine includes specific gravity, pH, urine sugar, protein, casts, ketone bodies, blood cells, and RBC.

g. Coagulation function includes PT, APTT, INR, FIB, TT, and D-dimer.

h. Thyroid function includes TSH, FT3, FT4, T3, T4. No relevant drug interference exists, and the test results within two weeks of the screening or baseline period are acceptable.

i. Adrenaline testing includes ACTH and cortisol. There is no interference from related drugs, and the test results are acceptable within two weeks of the screening or baseline period.

j. Blood glucose examination includes blood glucose and glycated hemoglobin. The glycated hemoglobin test results within 12 weeks during the screening and baseline periods are acceptable.

k. T-cell immune includes CD3^+^T cell count, CD4^+^T cell count, and CD8^+^T cell count.

**1 Background**

- 1. **Immune Escape Of Virus**

Various viral immune evasion mechanisms can result in persistent viral infections ^1-3^. For instance, the interaction between viruses and host immune cells can weaken the interferon signaling pathway, leading to dysfunction in interferon DCs, macrophages, and natural killer cells^1^. Additionally, the rapid selection of immune-escape variants can evade adaptive immune responses. T cells play a pivotal role in the process of clearing the virus. Prolonged antigen expression, induced by chronic immune activation, can lead to T cell exhaustion and dysfunction, further contributing to persistent viral infection^1,2,4,5^. An analysis of T cells in a mouse model of LCMV infection has demonstrated that T cell exhaustion is caused, at least in part, by the expression and function of inhibitory receptor PD-1^6^.

PD-1 cell surface receptors and their ligands, PD-L1 (B7-H1) and PD-L2 (B7-DC) belong to the CD28-B7 family of T cells and play a crucial role in resisting foreign pathogens and maintaining a delicate autoimmune balance^7-9^. PD-1 can be induced and activated in various immune cell subsets, including CD4^+^ and CD8^+^ T cells, NK cells, B cells, monocytes, and DCs. PD-L1 is expressed on multiple lymphocytes and peripheral cells, and its expression is induced by inflammatory cytokines, often associated with viral infections like interferons. The expression of PD-L2 is primarily limited to myeloid cells, including DCs^9,10^. The interaction between PD-1 and PD-L1 also contributes to T cell development and regulatory functions. Research data suggest that the PD-1 pathway is a major mechanism of immune evasion exploited by human tumors^11,12^. Multiple solid tumors have shown overexpression of PD-1, PD-L1, and PD-L2, leading to tumor-induced inhibition of T cell activation through the expression of tumor-specific antigens^13-15^. The role of the PD-1/PD-L1 interaction in tumor immune evasion implies that immune responses can be restored by inhibiting the PD-1 pathway.

The PD-1 signal pathway has been shown to play a role in human chronic viral infections associated with T cell exhaustion^7^. Persistent viremia is linked to the upregulation of PD-1 expression in virus-specific CD8^+^ T cells^16-19^. In patients with HIV, HBV, or HCV infection, enhanced PD-1 expression in T cells is associated with T cell exhaustion, characterized by reduced virus-specific proliferation capacity and decreased cytokine expression^16,17,20,21^. Blocking the PD-1 signal pathway can partially restore CD8^+^ T cell function^16,17,20,22-25^. Consistent with these findings, the treatment of SIV-infected macaques with anti-PD-1 antibodies not only led to a rapid expansion of SIV-specific CD8^+^ T cells, improving their functional characteristics in vivo, but also enhanced resistance against SIV, humoral immunity, and overall survival^26,27^. Similarly, blocking the PD-1 signal pathway in vivo enhanced the response of antiviral effector T cells and controlled LCMV infection in mice^6^. Therefore, the PD-1 pathway presents potential therapeutic targets and can be widely used to treat chronic viral infections. Other immunosuppressive agents involved in the immune response to chronic viral infections include CTLA-4, T TIM-3, and LAG-3) ^4,28-30^.

**1.2 Introduction To Trial Drugs**

The recombinant humanized PD-L1 single-domain antibody Fc fusion protein injection, collectively called ASC22 by Ascletis' internal development code, is a human PD-L1 protein. It is a recombinant fusion protein-drug designed to block the interaction between PD-L1 and its receptor, PD-1 protein.

The structure of the ASC22 protein molecule is a homodimer formed by two identical subunits (dAb-Fc) through inter-chain disulfide bonds (Figure 1). Each subunit comprises two domains: a dAb domain fused to a mutant human immunoglobulin crystallizable region (Fc) domain.

The dAb component of ASC22 is a humanized VHH fragment that recognizes explicitly the human PD-L1 protein. Its amino acid sequence was identified through screening the immune library of the Xinjiang Bactrian camel (Camelus bactrianus) to isolate a VHH candidate sequence capable of effectively and explicitly blocking the interaction between human PD-L1 and PD-1 proteins. This sequence was further refined through humanization processes.

The mutated Fc domain sequence of ASC22 comprises the hinge region of intact human IgG1, the CH2 region, and the CH3 region of the heavy chain constant region. Concurrently, mutations were introduced to the Fc fragment of human IgG1 to eliminate Fc-mediated ADCC and CDC activities^5,28^. Furthermore, as ASC22 lacks a light chain sequence, the Cys typically used to form intermolecular disulfide bonds with the delicate chain in the hinge region was replaced with a Ser.


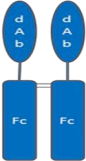


Theoretical molecular weight: 79563.8 Da

Actual molecular weight: about 82-84 Da

**Figure1 Schematic diagram illustrating the composition of the ASC22 protein molecule.**

In November 2020, ASC22, known as KN035 and marketed as envolimumab injection, submitted a NDA for marketing authorization to the State Drug Administration. It was officially accepted on December 17, 2020, under the acceptance number CXSS2000060, and is currently undergoing technical review.

Indications for ASC22 include treating MSI-H advanced colorectal cancer in patients who have previously received fluorouracil, oxaliplatin, and irinotecan. MSI-H advanced gastric cancer in patients who have previously failed at least one line of standard therapy. Advanced solid tumors in patients with dMMR who have not responded to first-line standard treatment.

**1.3 Preclinical Studies**

**1.3.1 Pharmacokinetics**

After a single subcutaneous administration of ASC22 in cynomolgus monkeys at doses of 5, 15, and 50 mg/kg, both the peak concentration (Cmax) and plasma exposure [AUC(0-t)] increased proportionally with the dose escalation in a 1:3:10 ratio. The average Cmax ratios were 1:4.13:12.53, and the average AUC (0-t) ratios were 1:3.24:12.01. ASC22 exhibited linear kinetic characteristics during metabolism in cynomolgus monkeys, and there were no significant gender-related differences in the main pharmacokinetic parameters. The average absolute bioavailability of ASC22 after subcutaneous administration was 104.62%. However, the half-life of ASC22 after a single subcutaneous administration was significantly shorter in the low and middle dose groups (low dose group: t1/2=31.30±24.85 h; middle dose group: t1/2=26.48±13.59 h) compared to the high dose group (t1/2=155.81±29.33 h). This difference may be attributed to immunogenic-positive animals in the low and middle-dose groups. After a single subcutaneous administration of ASC22 at doses of 5, 15, and 50 mg/kg, the immunogenicity positivity rates (D37) were 5/6, 4/6, and 0/6, respectively. Additionally, after intravenous administration of ASC22 at 15 mg/kg, the original positivity rate was 6/6.

In a multiple-dosing study conducted in cynomolgus monkeys, the dosing regimen consisted of once-weekly administrations for five doses, followed by a 4-week recovery period. The doses administered were 0, 5, 30, and 150 mg/kg. Following the first and last administrations, the average Cmax and AUC (0-t) of ASC22 in each dose group increased in response to the escalating dose. Notably, no significant accumulation was observed between the first and last administrations in male and female animals across the low, middle, and high dose groups. Only animals in the low-dose group tested positive for anti-drug antibodies after the dosing period and at the end of the recovery period, with positivity rates of 4/10 and 3/4, respectively.

**1.3.2 Toxicology**

A multiple-dose toxicity study in cynomolgus monkeys was conducted using doses of 0, 5, 30, and 150 mg/kg administered via subcutaneous injection once a week for five doses, with a subsequent 4-week recovery period. No animals succumbed during the experiment. Various assessments, including clinical observations, food consumption, body weight, ophthalmological examinations, body temperature, ECG, blood pressure measurements, urinalysis, hematology, blood biochemistry, blood coagulation tests, and organ weight measurements, were performed. These assessments revealed no abnormal changes associated with the administration of ASC22. Serum thyroid function hormone detection showed that, except for TSH detection, most sample values were below the lower limit of quantification due to method limitations, and no valid data were obtained. There were no abnormal changes observed in T3 and T4 levels. Autopsy and microscopic examinations also revealed no abnormal changes related to administering the test product.

Toxicokinetic results indicated that within the dose range of 5-150 mg/kg, the average Cmax and AUC(0-t) of subcutaneously administered ASC22 in cynomolgus monkeys increased with the dose, with no significant accumulation observed in either male or female animals. At the end of the administration period, the ADA (anti-drug antibody) positivity rates for the ASC22 low, middle, and high dose groups were 4/10, 0/10, and 0/10, respectively. At the end of the recovery period, these rates were 3/4, 0/4, and 0/10, respectively.

In summary, the maximum NOAEL in this experiment was greater than 150 mg/kg/week.

**1.3.3 Pharmacodynamics**

**1.3.3.1 Anti-HIV Infection**

In PLWH, increased PD-1 expression on T cells is associated with T cell exhaustion, characterized by diminished virus-specific proliferation capacity and reduced cytokine expression. Medications targeting PD-L1 obstruct PD-1/PD-L1 signaling, alleviating the body's suppressive impact on T cells via the PD-1/PD-L1 signaling pathway and potentially facilitating the secretion of antiviral cytokines to achieve HIV clearance. The mechanism of action of ASC22 involves high-affinity binding to hPD-L1 through its antigen-binding region, competitively inhibiting the binding of PD-1 molecules to PD-L1, thereby promoting innate or adaptive immune responses and sustaining T cell activation.

Some researchers infected seven humanized mice (Rag-hu) with HIV-1 and subsequently administered subcutaneous injections of a PD-L1 monoclonal antibody ^8^. The results demonstrated that following the PD-L1 antibody injection, the serum HIV RNA load in the treatment group of mice continued to decrease. Compared to the control group, on Day 7, it had dropped by seven times; on Day 14, by 20 times; on Day 21, by 178 times; and on Day 28, by 269 times. In SIV infection experiments, the blockade of the PD-1/PD-L1 pathway was found to promote the expansion of CD4/CD8^+^ T cells, enhance cytokine secretion, boost SIV-related antibody production, promote IgG synthesis, and reduce SIV RNA levels^27^. Experiments involving the immune activation of isolated human peripheral blood cells revealed that the blockade of the PD-1/PD-L1 pathway can restore CD4^+^ T cell function and enhance the secretion of IL2 and IFN-gamma (both mRNA and protein levels)^31^. In clinical trials of anti-PD-1 antibody treatment for HIV infection, a single dose of anti-PD-1 antibody restored HIV-specific T cell function^32^.

Therefore, by inhibiting PD-1/PD-L1 signaling, the suppressive effect of the PD-1/PD-L1 pathway on T cells can be alleviated, immune system function can be restored, immune cell proliferation can be enhanced, and the secretion of antiviral factors can be promoted, ultimately leading to anti-HIV effects.

**1.3.3.2 Anti-HBV Infection**

This product is currently undergoing trials for chronic HBV infection. Standard non-clinical pharmacodynamic animal models used in chronic CHB antiviral therapy, such as the transgenic mouse model, the tail vein hypertensive injection mouse model, and the tree shrew HBV infection model, are better suited for drugs that directly target viral components, such as nucleoside (acid) drugs. Flow cytometry results indicate that KN035 does not recognize mouse PD-L1 molecules; therefore, these three models are unsuitable for drugs modulating the body's immune response, like PD-1/PD-L1 antibodies, due to species differences and immune resistance to antibody-based therapies.

According to ICH S6 guidance, surrogate antibodies can be used for animal experiments when a drug does not interact with its target in animal models. For instance, Ibalizumab (trade name: Trogarzo) does not exhibit strong binding activity with rhesus monkey CD4 molecules. Consequently, researchers opted for Mu5A8 (the mouse prodrug of Ibalizumab) for in vivo pharmacodynamic experiments, and it received FDA approval for treating multidrug-resistant HIV-1 infection in 2018.

In fact, the WHV is highly homologous to HBV, and both share similar PD-1/PD-L1 immune regulation mechanisms. Therefore, the Woodchuck model of WHV infection is currently considered the most suitable model for evaluating the efficacy of PD-L1 antibodies in the treatment of CHB.

Based on the reasons above, the researchers utilized the woodchuck PD-L1 antibody as a substitute for KN035 in non-clinical efficacy experiments. The results from animal experiments conducted on one of the WHV chronically infected woodchuck models revealed the following:

(1) In the group treated with ETV alone, the WHV DNA load tended to rebound. However, in the combined treatment group with woodchuck PD-L1 antibody, the WHV DNA level decreased by more than 6 logs. It remained at a low level 14 weeks after the conclusion of treatment, reaching the lower detection limit.

(2) In the prairie dog PD-L1 antibody combination treatment group, WHsAg levels decreased from 698.3 mg/ml at baseline to below 80 mg/ml, and this reduced level was sustained for 14 weeks after treatment cessation.

(3) The intrahepatic WHV DNA level in the PD-L1 antibody treatment group was significantly lower than in the non-PD-L1 antibody treatment group (ranging from 1.34×10^10^ to 4.33×10^10^ GE/ml vs. 0 to 1.0×10^8^).

(4) Southern blot results indicated that the WHV cccDNA level decreased solely in the PD-L1 antibody group, with no cccDNA detected in one case. Another animal experiment employing the same model yielded similar results, demonstrating that the combination therapy of PD-L1 antibody and ETV can effectively and sustainably inhibit WHsAg, as indicated by the animal experiment results.

**2 Study Purpose**

Assessing whether ASC22 in combination with chidamide can reduce HIV reservoirs in PLWH with virologically suppressed

**3 Study Design**

**3.1 Basis For Dose Selection**

**3.1.1 Phase I Pharmacokinetic Study**

Phase I studies of ASC22 for tumor indications have received approval in the United States, China, and Japan, respectively. Phase I clinical trials were conducted in 2016 or 2017 with the primary objective of assessing the safety, tolerability, and pharmacokinetic characteristics of ASC22. The study design included a total of 8 dose groups: 0.01 mg/kg, 0.03 mg/kg, 0.1 mg/kg, 0.3 mg/kg, 1.0 mg/kg, 2.5 mg/kg, 5.0 mg/kg, and 10.0 mg/kg, with dosing once a week.

Phase I studies of ASC22 for tumor indications have received approval in the US, China, and Japan, respectively. Phase I clinical trials were conducted in 2016 or 2017 with the primary objective of assessing the safety, tolerability, and pharmacokinetic characteristics of ASC22. The study design included a total of 8 dose groups: 0.01 mg/kg, 0.03 mg/kg, 0.1 mg/kg, 0.3 mg/kg, 1.0 mg/kg, 2.5 mg/kg, 5.0 mg/kg, and 10.0 mg/kg, with dosing once a week.

Currently, pharmacokinetic data for ASC22 in some studies in China and Japan have been collected, revealing the following characteristics. The plasma concentration of ASC22 increases with the dose, and there is no significant variation among participants within the same dose group in the plasma concentration and elimination curve. During repeated dosing, evident drug accumulation is observed from the first to the fifth dose. By the fifth dose, a steady-state concentration of the drug has yet to be reached, and the trend continues to rise. The average elimination half-life of ASC22 in subjects is approximately 200 hours or 8.3 days.

**Table 2: Pharmacokinetic Summary of Phase I Clinical Study of ASC22**

| Cycle-day | Time(h) | Dose(mg/kg ，QW) | | | | Dose(mg/kg ，Q2W) |
| --- | --- | --- | --- | --- | --- | --- |
|  |  | 0.3 | 1 | 2.5 | 5 | 5 |
|  |  | C(ng/ml) | | | | C(ng/ml) |
| C1-d1 | 0 | 0 | 0 | 0 | 0 | 0 |
| C1-d1 | 2 | 0 | 233 | 1 **Viral** **Suppression** | 207.4 |  |
| C1-d1 | 4 | 0 | 312 | 266 | 1072 |  |
| C1-d1 | 8 | 203 | 650 | 844 | 4047 |  |
| C1-d1 | 12 | 299 | 1073 | 1378 | 8047 |  |
| C1-d2 | 24 | 366 | 1822 | 2540 | 14240 | 6043.3 |
| C1-d3 | 48 | 512 | 2449 | 4773 | 22333 | 10937.6 |
| C1-d4 | 72 | 495 | 2690 | 5053 | 23833 | 18908.3 |
| C1-d5 | 96 | 482 | 2610 | 5155 | 21967 | 20146.7 |
| C1-d6 | 120 | 365 | 2773 | 5090 | 20100 | 20266.7 |
| C1-d8 | 0 | 380 | 3137 | 4788 | 19267 | 18866.7 |
| C1-d15 | 0 |  | | | | 14360 |

**3.1.2 Effectiveness Research**

**3.1.2.1 Anti-HBV Efficacy**

The ASC22 phase IIa clinical trial that has been conducted is a single-dose escalation study using three subcutaneous doses (0.3, 1.0, and 2.5 mg/kg, each dose injected to three patients) with 12 weeks of follow-up to explore the safety and efficacy of ASC22 in patients with CHB (ClinicalTrials.gov number: NCT04465890). The primary efficacy endpoint of the trial is the reduction of HBsAg at the 12-week follow-up after a single dose. Nine patients enrolled in the study with CHB had a median baseline HBsAg level of 3.0 log_10_ IU/mL.

Data from this Phase IIa clinical trial demonstrated a dose-dependent decrease in HBsAg after a single dose of ASC22 (0.3, 1.0, and 2.5 mg/kg, respectively). Eight of nine ASC22-treated patients showed some reduction in HBsAg at the end of the 12-week follow-up. One of three patients receiving the 2.5 mg/kg dose had a maximum decrease in HBsAg of 1.2 log10 IU/mL at 12 weeks of follow-up.

**3.1.2.2 Anti-tumor Efficacy**

In Phase I studies conducted in China, the US, and Japan, among the 35 subjects who received at least one efficacy evaluation after administering the study drug, there were four PRs, 12 cases of SD, and 19 cases of PD. These results suggest that ASC22 demonstrates significant anti-tumor efficacy within the dose range of 0.01 mg/kg to 10 mg/kg.

**Table 3 The effectiveness of ASC22**

| **NO.** | **Stages** | | **Indications** | **PR (Dose/number of participants/treatment cycles)** | **SD (Dose/number of participants/treatment cycles)** |
| --- | --- | --- | --- | --- | --- |
| ASC22-US- 001 | Phase I | | Advanced solid tumors | 0.3mg/kg, 1, 8, 9  2.5mg/kg, 1, >5 | 0.3 mg/kg, 1, 8.3  1.0 mg/kg, 1, 8.3  5.0 mg/kg, 1, >4 |
| ASC22-CN- 001 | Phase I | Dose escalation | Advanced solid tumors | 2.5mg/kg, 1, >7.5 | 1.0 mg/kg, 2, (7, >9)  2.5 mg/kg, 2, (>7, >6)  5.0 mg/kg, 3, (>4,>4)* |
|  |  | Dose expansion 1 | Advanced hepatocellular carcinoma | 2.5mg/kg, 2 | 2.5 mg/kg, 7  5.0 mg/kg, 3 |
|  |  | Dose expansion 2 | Advanced solid tumors | 1 |  |
| ASC22-JP- 001 | Phase I | | Advanced solid tumors | 1.0 mg/kg, 1, 7 | 1.0 mg/kg, 1, 6  2.5 mg/kg, 1, 3  5.0 mg/kg，  Evaluation time has not yet arrived* |

*Until May 15, 2018

Until October 2018, Ascletis Pharma Inc., China selected two doses of 2.5 mg/kg and 5.0 mg/kg and conducted safety and tolerability assessments of ASC22 in 40 liver cancer subjects using a weekly dosing regimen. Currently, the maximum treatment duration in this group is nine months. Two participants (2/40) observed liver abnormalities, resulting in grade 3 liver injury and grade 4 liver bleeding. However, it's important to note that both cases were attributed to disease progression. Therefore, up to the present moment, the administration of ASC22 at 2.5 mg/kg and 5.0 mg/kg once a week has been well-tolerated in participants with liver cancer, and no instances of drug-related liver dysfunction or injury have been observed.

In the phase II clinical trial (NCT03667170) evaluating KN035 monotherapy for treating MSI-H/dMMR advanced solid tumors, a single-arm open-label design was employed to study the primary endpoint. The BIRC assessed the confirmed ORR. MSI-H status for CRC and GC was verified through central pathology, while dMMR status for other tumor types was assessed by local pathology. A total of 103 patients were enrolled in the study. The confirmed ORR set by BIRC in the overall population (n=103) was 42.7%. In CRC patients (n=65) and GC patients (n=18), the ORR was 43.1% and 44.4%, respectively. For patients with other solid tumors (n=20), the ORR was 40.0%. The median DOR assessed by BIRC in the overall population was not reached, with a 12-month DOR rate of 92.2%. The median PFS was 11.1 months, and the median OS was not reached, with an OS of 12-month rate of 74.6%.

**3.1.3 Security**

Until May 2, 2018, participants who completed the DLT assessment in the 0.01-10 mg/kg dose groups of the Phase I anti-cancer studies conducted in the US and China had no experienced DLTs. Similarly, no DLTs occurred in any paticipants who completed the DLT assessment in the Japanese Phase I study's 1.0-5.0 mg/kg dose group. Drug-related AEs observed across these studies were primarily of grade 1-2. There was no positive correlation between the incidence of study drug-related adverse events and the administered drug dose. Notably, only one drug-related grade 3 or higher AEs occurred in the studies in China and the US. The study in China showed grade 3 autoimmune dermatitis in the 0.3 mg/kg dose group, which resolved after 33 days of drug intervention. The study in the US showed grade 3 lymphopenia in the 0.1 mg/kg dose group, which was decided after one week without drug intervention. In summary, ASC22 demonstrated a high level of safety and good tolerability among all enrolled subjects in the 0.01-10 mg/kg dose group.

ASC22 has conducted a Phase IIa/IIb clinical study in CHB. Phase IIa comprised a single-dose escalation involving three dose groups of 0.3 mg/kg, 1.0 mg/kg, and 2.5 mg/kg, each with three participants. On the other hand, Phase IIb is a multiple-dose study, with selected dose groups of 1.0 mg/kg and 2.5 mg/kg. Each group receives 12 administrations, administered once every two weeks. The Phase IIa clinical study was completed on June 7, 2021, with participants exhibiting good safety profiles, and all AEs were of grade 1. In the Phase IIb study, 74 participants were enrolled in the 1.0 mg/kg group, and 48 participants were enrolled in the 2.5 mg/kg group. It is currently ongoing for Phase IIb study.

In the phase II pivotal clinical trial (NCT03667170) evaluating KN035 monotherapy for the treatment of MSI-H/dMMR advanced solid tumors, 16 out of the 103 enrolled patients (15.5%) experienced grade 3 to 4 drug-related TEAEs. There were no Grade 5 TEAEs related to the study drug. Approximately 2.9% of the subjects permanently discontinued treatment due to drug-related TEAEs. The incidence and occurrence of immune-related AEs were comparable to those observed with similar products. No cases of immune pneumonia or colitis were reported, and there were no instances of infusion reactions. The incidence of injection site reactions was 8.7%, all of which were of grade 1 to 2, with no related serious adverse events or events leading to permanent discontinuation.

**3.1.4 The Steady-state Target Concentration Of ASC22**

Based on the human pharmacokinetic test results of ASC22 at a dose of 5 mg/kg administered every two weeks and its predominantly linear pharmacokinetic characteristics, it is suggested that a minimum amount of 1.0 mg/kg may be necessary to reach a steady-state target concentration of 3 μg/ml.

**3.2 Inclusion And Exclusion Criteria**

**3.2.1 Inclusion Criteria**

(1) Individuals diagnosed with HIV infection;

(2) Age ≥ 18 years old;

(3) BMI ≥ 18.0 to < 35.0 kg/m^2^;

(4) Capable and willing to comply with the time requirements for study visits and assessments;

(5) Currently receiving ART for at least 24 months, with two consecutive plasma HIV-1 RNA levels <50 copies/ml, separated by an interval of at least 12 months;

(6) During the screening period, CD4^+^ T cell count is ≥ 250 cells/µl (including the boundary value), and CD4/CD8 ratio is < 0.9;

(7) Agree to use contraception during participation in the project and for up to 6 months after the end of the trial;

(8) Willing to provide informed consent.

**3.2.2 Exclusion criteria**

(1) Those who have suffered from any serious acute illness within 8 weeks;

(2) Subjects with a history of active autoimmune disease or autoimmune disease requiring systemic treatment;

(3) Prior treatment/exposure to any other ICIs (e.g., PD-1, anti-PD-L1, anti-PD-L2, anti-CTLA4, etc.);

(4) Patients who have undergone the following treatments:

a) Received other anti-latent drug treatments within 30 days before enrollment;

b) Underwent radiotherapy or chemotherapy within 30 days before screening;

c) Received immunosuppressive therapy within 60 days before screening;

d) Received immunomodulatory agents (e.g., interleukins, interferons), hydroxyurea, or foscarnet within 60 days before screening;

e) Received an HIV vaccine or systemic cytotoxic chemotherapy within 60 days before screening;

f) Previously received IgG treatment;

g) Underwent blood transfusion or cell growth factor treatment within 90 days before screening;

h) Currently using or planning to use rifampicin, rifabutin, and other drugs during screening;

(5) Individuals whose laboratory examinations meet the following criteria:

a) ANC < 1.50× 10^9^/L; Hb < 105 g/L (for men) or < 95 g/L (for women); platelets < 75× 10^9^/μL; INR > 1× ULN;

b) SGPT/ALT > 1.5× ULN, SGOT/AST > 1.5× ULN, total bilirubin, direct bilirubin > 1.5× ULN, serum creatinine > 1.5× ULN, and the abnormalities are clinically significant;

c) Clinical significance of five thyroid function abnormalities, including T3, T4, FT3, FT4, and TSH;

d) Abnormal epinephrine test with clinical significance, including at least ACTH and cortisol;

e) Abnormal blood glucose and glycosylated hemoglobin tests with clinical significance;

(6) Abnormal and clinically significant findings on the twelve-lead electrocardiogram at the time of enrollment;

(7) Presence of interstitial changes on chest CT examination at the time of enrollment;

(8) Individuals with severe heart disease, symptomatic or asymptomatic arrhythmia;

(9) Patients co-infected with HBV, HCV, syphilis, other liver diseases, and diabetic patients;

(10) Individuals with active or suspected malignant tumors or a history of malignant tumors within the past five years (except for basal cell skin or cervical cancer in situ);

(11) Individuals with a history of tuberculosis or active tuberculosis;

(12) Subjects with psychiatric or substance abuse disorders known to interfere with their ability to meet study requirements;

(13) Individuals who have received immunomodulatory or immunosuppressive treatments (including any dose of IV/oral [PO] steroids, but excluding inhalation, topical, or local injection steroids) within 24 weeks before the first dose of the study drug;

(14) Pregnant and lactating women and individuals, both men and women, who plan to have children during the study;

(15) Individuals with mental health conditions or substance abuse issues that may interfere with the test;

(16) Patients who have used HDACI, such as valproic acid, butyrate, phenylbutyrate, etc., may be enrolled after a 28-day washout;

(17) Patients with severe cardiac insufficiency (New York Heart Association [NYHA] cardiac insufficiency class IV);

(18) Patients with a history of arterial thromboembolic events, including myocardial infarction, unstable angina; cerebrovascular accident, or transient ischemic attack, within six months before enrollment in treatment; standard treatment of uncontrolled hypertension (systolic blood pressure ≥ 150 mmHg or diastolic blood pressure ≥ 100 mmHg); and patients with cardiomyopathy;

(19) Patients with a significant QT/QTC interval prolongation during the screening period (e.g., repeated measurements show QTc interval > 450 milliseconds, or other conditions that may lead to TdP risk, such as heart failure; hypokalemia, familial long QT syndrome) or concurrent use of drugs that may prolong the QT/QTc interval;

(20) Individuals with known allergies or anti-drug antibodies to the drugs or excipients used in this trial;

(21) Individuals deemed unsuitable to participate in this trial by the researcher.

**3.2.3 Exit Criteria**

**3.2.3.1 Withdrawal At The Investigator's Discretion**

(1) During the clinical trial, if the investigator determines that the subject is unsuitable to continue participating in this study.

(2) In the event of serious AEs, and if the investigator determines that continuing the study will not be beneficial.

(3) In cases where subjects experience serious AEs and are deemed unsuitable to continue participating in the research.

(4) When, following treatment administration, the subject exhibits poor medication compliance in the absence of AEs.

(5) If the subject participates in other concurrent clinical trials during this study.

(6) When other drugs that may impact tolerance and pharmacokinetic analysis and judgment are used during the trial.

(7) In the case of an unexpected pregnancy.

3.2.3.2 Voluntary Withdrawal by Paticipants

If the participants don't wish to continue participating in the clinical research, they have the right to withdraw from the study at any stage without facing discrimination or retaliation, as outlined in the informed consent form. Not explicitly expressing a desire to exit but ceasing to take medication, undergo testing, and becoming lost to follow-up are also considered forms of "dropping out" or "withdrawal." Possible reasons for withdrawal may include:

(1) The subject withdraws their informed consent.

(2) Circumstances prevent the subject from continuing to complete follow-up assessments on time for various reasons.

**3.2.4 Post-Exit Procedures**

The investigator should meticulously document the reason and timing of the subject withdrawal, conduct appropriate observations and assessments for withdrawn subjects according to the study plan, and complete the specified evaluations after the retreat.

These details should be recorded in the corresponding source documents, accompanied by a description of the reason for suspension.

Participants who quit the study should continue to be monitored for AEs. Negative event follow-up for these subjects should persist until their condition returns to baseline or until the investigator determines that further observation is unnecessary. Throughout the study, all participants who received medication but failed to complete the prescribed research activities outlined in the protocol should have their last test results considered as the outcome. Investigators should proactively reach out to those patients who did not comply with the requirements and maintain accurate records of these contact attempts in the corresponding source documents (e.g., including the time and date of telephone contact, proof of registered mail receipt, etc.), ensuring proper documentation.

No further data collection is necessary in the event of withdrawal for the following reasons: (1) the participants withdraw their informed consent; (2) the participants experience a fatal event; (3) the study is terminated.

**3.2.5 Criteria for Terminating the Study**

Study termination may occur under the following circumstances:

(1) The subject experiences intolerable toxicity, and the investigator determines they are unsuitable to continue receiving the experimental drug.

(2) Significant errors are identified in the study protocol.

(3)The administrative department cancels the study.

Comprehensive suspension or termination of the study can be temporary or permanent. Please keep all study records for future reference in case of study suspension or termination.

**4 Drugs**

**4.1 Investigational drugs**

ASC22 is a monoclonal antibody drug for subcutaneous injection. The minimum packaging unit is a single-use glass vial made of brown neutral borosilicate, and each vial comes in a box. The drug is dosed at 1ml: 200 mg (1 ml/ampoule). ASC22 is manufactured by Alphamab Oncology, Jiangsu, China. It has been donated by Ascletis Pharma Inc., China. Storage conditions: Store between 2-8℃.

Chidamide tablets (Trade Name: Aipusha) are an HDAC inhibitor for oral administration. The drug specifications are 5 mg per tablet; each box contains 24 tablets. These tablets are manufactured by Shenzhen Chipscreen Biosciences, China, and have been provided free of charge by Ascletis Pharma Inc., China. Storage conditions: Keep in a light-shielding and sealed container store below 25°C.

**4.2 Antiviral regimen drugs**

ART treatment

**5 Clinical trial content**

**5.1 Sample size**

This study estimates that 12 participants will complete the trial, and the dropout rate will be controlled within 15%, so 15 participants are planned to be enrolled.

**5.2 Test Process**

This project is a prospective, single-center, single-arm clinical trial study. The plan is to enroll 15 PLWH. Following informed consent, each subject will receive a single dose of 1 mg/kg intravenous ASC22 on the first day, followed by a subcutaneous injection of ASC22 (drawing an appropriate volume of ASC22 according to the participant's needs and then injecting subcutaneously, with the injection speed not exceeding 0.06 ml/s). Additionally, oral chidamide intervention will be administered twice a week simultaneously, with a dosage of 10 mg.

Throughout this period, the ART treatment will be continued. The treatment period will last 12 weeks, and follow-up will extend until 24 weeks. Peripheral blood samples will be collected before the ASC22 intervention(every four weeks). Approximately 20 ml of blood will be collected during each session, and subjects will undergo medical evaluation concurrently.

**5.3 Flowchart Of The Study**


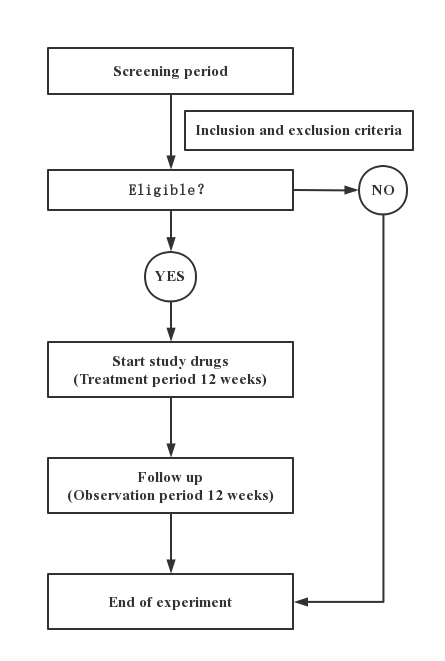


**Figure 2 ASC22 clinical trial flow chart**

**5.4 Test Result**

**5.4.1 Security Monitoring**

The related symptoms and laboratory tests need to be monitored. These symptoms may include vomiting, fatigue, rash, itching, diarrhea, constipation, borborygmi, fever/chills, headache, palpitations, heat sensation, changes in appetite, gastrointestinal pain, gastroesophageal reflux, touch sensitivity, taste and smell disturbances, dizziness, muscle or joint pain, blisters, oral lesions, conjunctivitis, facial edema, and more.

The severity of AEs will be graded according to the CTCAE version 6.0. AEs are defined as any adverse medical events occurring in patients or clinical research participants taking a drug product, regardless of causality with the treatment. The investigator will assess the relationship of each event to the study drug as either reasonably likely or not reasonably likely. AEs are divided into grades 1-5, with grade 3 being serious but not life-threatening and requiring hospitalization, grade 4 being life-threatening and requiring hospitalization, and grade 5 indicating an AE-related death requiring immediate intervention.

**5.4.2 The Activation of HIV Reservoir Detection**

a. Plasma HIV RNA at Different Follow-Up Points: Detection and evaluation are based on the quantitative detection kit of human immunodeficiency virus nucleic acid.

b. HIV-1 RNA in cells at 24 Weeks: PBMCs are isolated from peripheral blood. Following cell lysis and intracellular viral RNA extraction, the RNA is reverse-transcribed into cDNA. HIV-1 RNA is detected in PBMCs by RT-PCR.

c. Changes in the function of HIV Gag-specific CD8^+^T cells.

**5.4.3 The changes of HIV reservoir**

Digital droplet PCR is used to detect HIV-1 DNA in cells at various visit points (copies/106 PBMCs). PBMCs are isolated from 10ml whole blood samples. The QX100 digital droplets PCR system (Bio-Rad) is utilized to quantify the amount of HIV-1 DNA per 10^6^ PBMCs.

**5.4.4 Immunological Examination**

CD4^+^T cell count, CD8^+^T cell count, CD4^+^/CD8^+^ ratio

**5.4.5 Laboratory Examination and Organ Function Evaluation**

During the comprehensive assessment and clinical observation of subjects' relevant laboratory test indicators and organ functions based on the follow-up time, the following abnormal indicators will be focused on at the specified intervals:

(1) Vital Sign Monitoring: During each follow-up visit, BMI index, body temperature, blood pressure, heart rate, etc., will be checked to assess the occurrence of abnormalities.

(2) Cardiac Function Test: During each follow-up visit, electrocardiogram, myocardial enzyme spectrum, etc., will be examined. Cardiac ultrasound examinations will be conducted during the screening period, after using the experimental drug at week six, and upon completion of the treatment.

(3) Liver and Kidney Examination: During each follow-up visit, blood biochemistry, including liver and kidney function, will be examined. Abdominal B-ultrasound will be conducted during the screening period, after using the experimental drug at week six, and upon completion of the treatment.

(4) Respiratory System Examination: Chest CT examinations will be performed during the screening period, after using the experimental drug at week six, and upon completion of the treatment.

(5) Other Relevant Examinations: At each follow-up, blood routine, urine routine, coagulation function, electrolytes, etc., will be checked accordingly.

Researchers need to comprehensively evaluate the examination above results of the subjects based on the relevant physical reviews conducted at each follow-up visit. If abnormalities are detected in the appropriate tests during the study, timely adjustments should be made based on the clinical situation.

**6 Efficacy and safety outcomes**

The primary outcomes were assessed by quantifying changes in CA HIV RNA, total HIV DNA, integrated HIV DNA in PBMCs, and plasma HIV RNA, as well as by analyzing variations in the functionality of HIV Gag- and Pol- specific CD8^+^ T cells from baseline to post-treatments. In addition, changes in CD4^+^ T cell counts, CD8^+^ T cell counts, and CD4/CD8 ratios were monitored at baseline and at different time points during treatment.

Safety Outcomes: number of participants experienced with drug-related AEs of Grade 3 or Higher.

**7 Research Monitoring, Auditing, and Inspection**

**7.1 Research monitoring**

If the confidentiality complies with local requirements, the responsible monitor (or designated person) will maintain regular contact with the investigator and conduct periodic visits, during which they will be allowed to inspect various trial records, including case report forms and other relevant data, as necessary. Throughout the study period, monitors are responsible for regularly reviewing case report forms, ensuring compliance with the study protocol, and verifying entered data's completeness, consistency, and accuracy. Monitors should also have access to laboratory test reports and other subject records to validate the information recorded on case report forms. The investigator (or their designee) agrees to cooperate with the monitors to address any issues identified during these monitoring visits.

**7.2 Research Auditing and Inspection**

After receiving formal notification, the researcher should prepare the original records related to the study and provide them to the corresponding qualified personnel, their designated representatives, and the health department inspectors. Data reconciliation in medical report forms must be conducted directly against the original records.

**8 Publication of Research Data**

The research findings may be published or presented at scientific conferences.

**9 Investigator Qualifications**

Investigators must possess the medical qualifications to conduct this study and have sufficient time to supervise the implementation of clinical trials at their research center. Researchers will be subject to study-related monitoring, auditing, EC review, and supervisory body inspections, granting them direct access to original data.

The researcher must also list the names of the participants in the trial in the "Authorization Form" and sign the form, specifying their respective responsibilities. This table should be updated when personnel responsibilities change.

**10 Ethical and Legal Issues**

The research process must adhere strictly to the declaration of Helsinki (2013 version) and GCP for drug clinical trials, and it should be conducted in accordance with the requirements outlined in this trial protocol.

**10.1 EC**

Before the start of the clinical trial, the research protocol, informed consent, and other materials provided to the subjects must undergo review and approval by the IEC of the hospital, and the relevant approval documents must be provided to the sponsor.

If this protocol needs to be revised during the actual implementation of the clinical trial, the modified trial protocol will be resubmitted to the ethics committee for approval before implementation. If significant new information about the experimental drug emerges, the informed consent form must be revised in writing and submitted to the ethics committee for approval. The subject's consent must be obtained again.

During the actual implementation of this protocol in clinical trials, if the researcher determines that the revised protocol can better safeguard the rights of the subjects, adjustments can be made promptly during the trial. The modified protocol should be submitted to the ethics committee for review and approval. In this case, the protocol is not considered to be in violation.

**10.2 Informed Consent**

The researcher is responsible for providing each patient with detailed and comprehensive information, both orally and in writing, regarding the study's purpose, process, potential risks, and possible benefits before enrolling them. Patients must also be informed of their right to decide whether to participate in the study and withdraw at any time without facing discrimination.

After carefully reading and fully understanding its contents, the patient or their legal representative should sign the informed consent form. They should also retain a copy of the signed page for their records.

**10.3 Confidentiality**

All records related to the participants' identities will be kept confidential, and this information will not be disclosed to the public to the extent permitted by relevant laws and regulations. Only personnel involved in the trial, such as researchers and research nurses, will have access to the identity information.

In the CRF, only the participants' numbers and initials will be recorded; the names of the participants will not be included. If the name appears on any other document (e.g., pathology report), it must be redacted on the copy of the document. Computer-stored research reports must comply with local data protection laws. The identities will also be kept confidential when the study results are published. The investigator may maintain a list of records to identify participants.

**11 Data storage**

After the conclusion of the study, all research materials will be retained, including the verification of all participating subjects (effectively checking various records such as CRE and original hospital records), all original signed patient consent forms, all CRFs, and detailed records of drug distribution, among others. These materials will be preserved for 5 years after the trial's conclusion.

**12 References**

1. Rehermann, B. Hepatitis C virus versus innate and adaptive immune responses: a tale of coevolution and coexistence. *J Clin Invest* **119**, 1745-1754 (2009).

2. Boutwell, C.L., Rolland, M.M., Herbeck, J.T., Mullins, J.I. & Allen, T.M. Viral evolution and escape during acute HIV-1 infection. *J Infect Dis* **202 Suppl 2**, S309-314 (2010).

3. Op den Brouw, M.L.*, et al.* Hepatitis B virus surface antigen impairs myeloid dendritic cell function: a possible immune escape mechanism of hepatitis B virus. *Immunology* **126**, 280-289 (2009).

4. Burke, K.P. & Cox, A.L. Hepatitis C virus evasion of adaptive immune responses: a model for viral persistence. *Immunol Res* **47**, 216-227 (2010).

5. Rehermann, B. Chronic infections with hepatotropic viruses: mechanisms of impairment of cellular immune responses. *Semin Liver Dis* **27**, 152-160 (2007).

6. Barber, D.L.*, et al.* Restoring function in exhausted CD8 T cells during chronic viral infection. *Nature* **439**, 682-687 (2006).

7. Hofmeyer, K.A., Jeon, H. & Zang, X. The PD-1/PD-L1 (B7-H1) pathway in chronic infection-induced cytotoxic T lymphocyte exhaustion. *J Biomed Biotechnol* **2011**, 451694 (2011).

8. Francisco, L.M., Sage, P.T. & Sharpe, A.H. The PD-1 pathway in tolerance and autoimmunity. *Immunol Rev* **236**, 219-242 (2010).

9. Sharpe, A.H., Wherry, E.J., Ahmed, R. & Freeman, G.J. The function of programmed cell death 1 and its ligands in regulating autoimmunity and infection. *Nat Immunol* **8**, 239-245 (2007).

10. Flies, D.B., Sandler, B.J., Sznol, M. & Chen, L. Blockade of the B7-H1/PD-1 pathway for cancer immunotherapy. *Yale J Biol Med* **84**, 409-421 (2011).

11. Berger, R.*, et al.* Phase I safety and pharmacokinetic study of CT-011, a humanized antibody interacting with PD-1, in patients with advanced hematologic malignancies. *Clin Cancer Res* **14**, 3044-3051 (2008).

12. Brahmer, J.R.*, et al.* Phase I study of single-agent anti-programmed death-1 (MDX-1106) in refractory solid tumors: safety, clinical activity, pharmacodynamics, and immunologic correlates. *J Clin Oncol* **28**, 3167-3175 (2010).

13. Latchman, Y.*, et al.* PD-L2 is a second ligand for PD-1 and inhibits T cell activation. *Nat Immunol* **2**, 261-268 (2001).

14. Brown, J.A.*, et al.* Blockade of programmed death-1 ligands on dendritic cells enhances T cell activation and cytokine production. *J Immunol* **170**, 1257-1266 (2003).

15. Dong, H.*, et al.* Tumor-associated B7-H1 promotes T-cell apoptosis: a potential mechanism of immune evasion. *Nat Med* **8**, 793-800 (2002).

16. Trautmann, L.*, et al.* Upregulation of PD-1 expression on HIV-specific CD8+ T cells leads to reversible immune dysfunction. *Nat Med* **12**, 1198-1202 (2006).

17. Peng, G.*, et al.* PD-1 upregulation is associated with HBV-specific T cell dysfunction in chronic hepatitis B patients. *Mol Immunol* **45**, 963-970 (2008).

18. Golden-Mason, L.*, et al.* Upregulation of PD-1 expression on circulating and intrahepatic hepatitis C virus-specific CD8+ T cells associated with reversible immune dysfunction. *J Virol* **81**, 9249-9258 (2007).

19. Shen, T.*, et al.* Characteristics and PD-1 expression of peripheral CD4+CD127loCD25hiFoxP3+ Treg cells in chronic HCV infected-patients. *Virol J* **8**, 279 (2011).

20. Day, C.L.*, et al.* PD-1 expression on HIV-specific T cells is associated with T-cell exhaustion and disease progression. *Nature* **443**, 350-354 (2006).

21. Maier, H., Isogawa, M., Freeman, G.J. & Chisari, F.V. PD-1:PD-L1 interactions contribute to the functional suppression of virus-specific CD8+ T lymphocytes in the liver. *J Immunol* **178**, 2714-2720 (2007).

22. Boni, C.*, et al.* Characterization of hepatitis B virus (HBV)-specific T-cell dysfunction in chronic HBV infection. *J Virol* **81**, 4215-4225 (2007).

23. Penna, A.*, et al.* Dysfunction and functional restoration of HCV-specific CD8 responses in chronic hepatitis C virus infection. *Hepatology* **45**, 588-601 (2007).

24. Nakamoto, N.*, et al.* Functional restoration of HCV-specific CD8 T cells by PD-1 blockade is defined by PD-1 expression and compartmentalization. *Gastroenterology* **134**, 1927-1937, 1937 e1921-1922 (2008).

25. Franceschini, D.*, et al.* PD-L1 negatively regulates CD4+CD25+Foxp3+ Tregs by limiting STAT-5 phosphorylation in patients chronically infected with HCV. *J Clin Invest* **119**, 551-564 (2009).

26. Finnefrock, A.C.*, et al.* PD-1 blockade in rhesus macaques: impact on chronic infection and prophylactic vaccination. *J Immunol* **182**, 980-987 (2009).

27. Velu, V.*, et al.* Enhancing SIV-specific immunity in vivo by PD-1 blockade. *Nature* **458**, 206-210 (2009).

28. Khaitan, A. & Unutmaz, D. Revisiting immune exhaustion during HIV infection. *Curr HIV/AIDS Rep* **8**, 4-11 (2011).

29. Zhang, Y.*, et al.* Tim-3 negatively regulates IL-12 expression by monocytes in HCV infection. *PLoS One* **6**, e19664 (2011).

30. Nakamoto, N.*, et al.* Synergistic reversal of intrahepatic HCV-specific CD8 T cell exhaustion by combined PD-1/CTLA-4 blockade. *PLoS Pathog* **5**, e1000313 (2009).

31. Porichis, F.*, et al.* Responsiveness of HIV-specific CD4 T cells to PD-1 blockade. *Blood* **118**, 965-974 (2011).

32. Gay, C.L.*, et al.* Clinical Trial of the Anti-PD-L1 Antibody BMS-936559 in HIV-1 Infected Participants on Suppressive Antiretroviral Therapy. *J Infect Dis* **215**, 1725-1733 (2017).
